# Systematic analysis of the transcriptome in small‐cell carcinoma of the oesophagus reveals its immune microenvironment

**DOI:** 10.1002/cti2.1173

**Published:** 2020-10-05

**Authors:** Qi Zhao, Yan‐Xing Chen, Qi‐Nian Wu, Chao Zhang, Min Liu, Ying‐Nan Wang, Yan‐Fen Feng, Jia‐Jia Hu, Jian‐Hua Fu, Hong Yang, Jing‐Jing Qi, Zi‐Xian Wang, Yun‐Xin Lu, Hui Sheng, Ze‐Xian Liu, Zhi‐Xiang Zuo, Jian Zheng, Jing‐Ping Yun, Jin‐Xin Bei, Wei‐Hua Jia, Dong‐Xin Lin, Rui‐hua Xu, Feng Wang

**Affiliations:** ^1^ State Key Laboratory of Oncology in South China Collaborative Innovation Center for Cancer Medicine Sun Yat‐sen University Cancer Center Guangzhou China; ^2^ Department of Pathology Sun Yat‐sen University Cancer Center State Key Laboratory of Oncology in South China Collaborative Innovation Center for Cancer Medicine Guangzhou China

**Keywords:** immune microenvironment, immunotherapy, small‐cell carcinoma of the oesophagus, transcriptome analysis

## Abstract

**Objectives:**

Although the genomic landscape of small‐cell carcinoma of the oesophagus (SCCE) has been dissected, its transcriptome‐level aberration and immune microenvironment status are unknown.

**Methods:**

Using ultra‐deep whole transcriptome sequencing, we analysed the expression profile of nine paired SCCE samples and compared the transcriptome with public transcriptomic data set of normal oesophageal mucosa and other cancer types. Based on the transcriptome data, the immune signatures were investigated. The genomic data of 55 SCCE samples were also applied for immune checkpoint blockade therapy (ICBT) biomarker evaluation including microsatellite instability (MSI) status, tumor mutation burden (TMB) and neoantigen burden (TNB). Also, we evaluated the CD8, CD68 and programmed death‐ligand 1 (PD‐L1) in 62 retrospective SCCE samples with IHC assay.

**Results:**

Differential expression analysis revealed that the cell cycle, p53, and Wnt pathways are significantly deregulated in SCCE. Immune microenvironment analysis showed that high leucocyte infiltration and adaptive immune resistance did occur in certain individuals, while the majority showed a relatively suppressive immune status. Immune checkpoints such as CD276 and LAG‐3 were upregulated, and higher M2 macrophage infiltration in tumor tissues. Furthermore, normal tissues adjacent to the tumors of SCCE presented a more activated inflammatory status than tumor‐free healthy controls. These observations showed that ICBT might benefit SCCE patients. As the critical biomarker of ICBT, TMB of SCCE was 3.64 with the predictive objective response rate 13.2%, while the PD‐L1‐positive rate was 43%.

**Conclusions:**

Our study systematically characterized the immune microenvironment in small‐cell carcinoma of the esophagus and provided evidence that several patients with SCCE may benefit from immune checkpoint blockade therapy.

## Graphical abstract

In this study, transcriptomic aberrance and immune microenvironment of small‐cell carcinoma of the esophagus (SCCE) was comprehensively characterized with transcriptome and immunohistochemistry analysis. Assessment of immune checkpoint blockade (ICB) treatment biomarkers provides a rationale for use of ICB treatment in patients with SCCE.

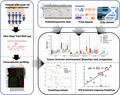

## Introduction

Small‐cell carcinoma can occur in virtually every organ, with the lung being the most common followed by the oesophagus.^1^, ^2^, ^3^ Small‐cell carcinoma of the oesophagus (SCCE) accounts for approximately 1–2.8% of all oesophageal carcinomas and is frequently diagnosed at late stages with positive neuroendocrine markers.^1^, ^3^ More than half of all SCCE patients present with distant metastases, leading to a median survival of only 8 to 13 months.^1^ There are no standard treatments for SCCE. The histopathology of SCCE is similar to those of small‐cell lung cancer (SCLC).^4^ Therapeutic strategies targeting SCLC have been widely employed for SCCE. Whether surgery could confine SCCE is still controversial, and most patients in the United States are not treated with surgery. Chemotherapy instead is effective in the initial treatment of SCCE and may improve disease outcomes.^4^ However, recurrence arises rapidly in the majority of patients, usually killing the individuals within a few months.^4^ Therefore, SCLC treatments are not optimal for patients with SCCE. In addition, recent evidence suggests that small‐cell carcinoma originating from different sites may be wildly distinct.^4^

Immune therapies, especially immune checkpoint blockade and chimeric antigen‐receptor (CAR) T‐cell therapies, have shown exciting long‐term efficacy in several cancers.^5^, ^6^ Immune checkpoint inhibitors targeting PD‐1 or PD‐L1 have taken over conventional treatments as the first‐line therapy for advanced several cancer types.^7^, ^8^ However, the response rates of patients are limited due to several factors influencing the efficacy of immunotherapy, such as activation of the immune system, the expansion of effector cells, the infiltration of activated effector cells to the tumor tissue, and the destruction of tumor cells.^9^ Together with the positive expression of PD‐L1 in the tumor tissue, tumor‐infiltrating effector cells or leucocytes indicate an adaptive immune resistance microenvironment of the tumor, which indicates a potential positive response to immune checkpoint inhibitors.^10^

The solid tumor microenvironment includes stroma cells, inflammatory cells, vasculature, extracellular matrices and tumor cells themselves. The tumor microenvironment usually suppresses the infiltration of lymphocytes and other effector cells, resulting in a mitigated antitumor response of the host. An accurate evaluation of the tumor microenvironment is an effective way to predict the response of immunotherapy and to avoid unnecessary treatments or possible side effects. Several biomarkers, such as tumor mutation burden (TMB), PD‐L1 expression, microsatellite instability and tumor neoantigen‐related mutation burden (TNB), can be used to screen for patients who can benefit the most from immunotherapy.

Thus far, immunotherapy has given hope to patients with advanced cancers without standard treatments. Unfortunately, there is no evidence to support testing immune treatments for SCCE. To explore the potential of immune checkpoint inhibitors on SCCE, we systematically investigated the transcriptomes and microenvironment of SCCE by using RNA sequencing data from nine pairs of frozen samples. With genomic aberrance we reported previously, transcriptomic profiling revealed several significant molecular events that might contribute to the tumorigenesis and development of SCCE. The individual examination of the immune microenvironment showed that adaptive immune resistance does occur in some cases in the tumor tissue of SCCE, while most present a relatively suppressive immune microenvironment in tumor tissues versus NATs, with the upregulation of several immune inhibitory factors. Moreover, by examining the transcriptome data of healthy oesophageal mucosa from the GTEx database, we found that the NATs of SCCE presented an abnormal state, showing an activating immune phenotype against healthy tissues, which indicated that targeting the upregulated immune inhibitory factors in tumor tissues might help reverse the suppressive immune microenvironment. Finally, we systematically assessed several therapeutic efficacy biomarkers of ICB, including microsatellite instability (MSI) status, TMB, neoantigen burden and PD‐L1‐positive rate, in SCCE with available whole‐exome sequencing data and pathologic analyses and revealed the possibility of effective immunotherapy in some SCCE patients.

## Results

### Samples and clinical data

Permission for the study was obtained from the Ethics Committee of Sun Yat‐Sen University Cancer Center. With written informed consent, fresh tumors and matched normal tissues adjacent to tumors (NATs) were collected from nine SCCE patients before any treatment. Detailed characteristics of the patients are summarised in Table 1.

**Table 1:** Clinical characteristic of patients with SCCE sequenced in this study

Patient ID	Patient 1	Patient 2	Patient 3	Patient 4	Patient 5	Patient 6	Patient 7	Patient 8	Patient 9
Gender	Male	Male	Male	Male	Female	Male	Male	Male	Male
Age at diagnosis	55–60	60–65	55–60	60–65	70–75	45–50	50–55	55–60	65–70
Smoking status	Y	N	Y	Y	N	Y	Y	Y	Y
Drinking status	Never	Never	Regular	Never	Never	Regular	Never	Never	Never
Family history	N	N	N	N	N	Y	N	N	N
TNM status	pT3N3M0	pT2N0M0	pT2N0M0	pT1N1M0	pT2N0M0	pT4N1M0	pT3N3M0	pT4N1M0	pT3N0M0
Tumor stage	Ⅲ	Ⅱ	Ⅱ	Ⅱ	Ⅱ	Ⅲ	Ⅲ	Ⅲ	Ⅱ
Tumor location	Midthoracic	Midthoracic	Lower‐thorax	Midthoracic	Midthoracic	Mid‐lower‐thorax	Mid‐lower‐thorax	Lower‐thorax	Midthoracic
Primary Tumor/Metastasis	Primary tumor	Primary tumor	Primary tumor	Primary tumor	Primary tumor	Primary tumor	Primary tumor	Primary tumor	Primary tumor
Tissue source	Surgical resection	Surgical resection	Surgical resection	Surgical resection	Surgical resection	Surgical resection	Surgical resection	Biopsy	Surgical resection
NEO‐ADJUVANT chemotherapy (Yes/No)	No	No	No	No	No	No	No	No	No
Chemotherapy (Yes/No)	No	No	Yes	Yes	NA	Yes	Yes	No	No
Radiation (Yes/No)	No	No	No	No	NA	No	No	No	No
Survival status at last follow‐up	Dead	Dead	Dead	Dead	NA	Dead	Dead	Dead	Dead
Survival time (month)	5	7	18	3	NA	56	43	12	21

### Transcriptomic profiling of SCCE

**Figure 1:**
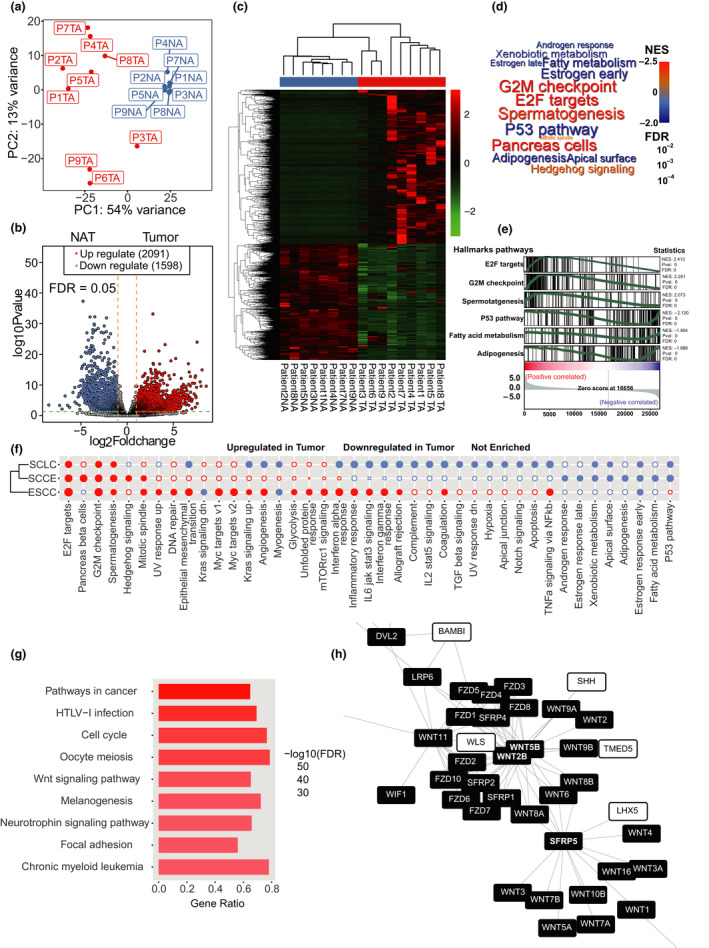
Transcriptomic profiling of SCCE. **(a)** Principal component analysis (PCA) plot for transcriptome data of 9 tumor tissues and 8 normal tissues adjacent to the tumor (NATs). **(b)** Volcano plot for differentially expressed genes (DEGs) in SCCE versus NAT. 2091 genes were upregulated while 1598 genes were downregulated in tumor against NAT. **(c)** Hierarchical clustering of the samples with expression level of DEGs. **(d)** Wordcloud plot for enriched Cancer Hallmark pathways (FDR < 0.05) in transcriptomic comparison between tumor tissues and NAT (red means activated while blue means suppressed in SCCE tissues against NATs). **(e)** GSEA plot for top 6 deregulated pathways in SCCE tissues versus NATs. **(f)** Hierarchical clustering of SCCE, small‐cell lung cancer (SCLC) and oesophageal squamous cell carcinoma (ESCC) based on deregulated pathways (a solid circle means FDR less than 0.05 while hollow circle means FDR larger than 0.05; circle size was in direct proportional to absolute value of normalised enrichment score (NES)). **(g)** Extended network constructed by NetworkAnalyst with top 1000 DEGs (500 upregulated and 500 downregulated genes ranked by fold change). Genes in the Wnt pathway are labelled. **(h)** Top 8 enriched pathways (ranked by FDR) in KEGG pathway enrichment analysis of genes in the extended network.

To avoid the underestimation of genes with low abundance because of an inadequate sequencing depth,^11^ we performed ultra‐high‐depth RNA sequencing on all available specimens, with a mean reads count of 49.99 Mb for tumor samples and 50.70 Mb for normal controls adjacent tissue (hereafter referred to as NATs). A series of quality evaluation steps ensured satisfactory sequencing libraries. Correlation analysis of tumor samples and corresponding NATs indicated that the NAT from patient 6 was aberrant and was therefore removed from the following analyses (Supplementary figure 1a). Principal component analysis of the transcriptomic profiles distinguished between tumor samples and NATs (Figure 1a). Unpaired differential expression analysis identified 2,091 upregulated and 1598 downregulated genes, with criteria of an adjusted *P*‐value < 0.05 and a fold change >2 (Figure 1b, c, Supplementary table 1). Gene set enrichment analysis (GSEA) (Supplementary table 2) showed that E2F targets, pancreatic beta cells, G2/M checkpoint and spermatogenesis were the most enriched gene clusters in SCCE tumors compared to NATs (Figure 1d, e). In contrast, the P53 pathway, adipogenesis apical surface and fatty acid metabolism were the most suppressed pathways in the tumor samples (Figure 1d, e). Given that NATs usually contain higher fraction of stromal content (such as adipose tissue), deregulation of the normal metabolic pathways (e.g. adipogenesis apical surface, fatty acid metabolism) might also be attributed to the different composition of normal cells. GO analysis based on the differentially expressed genes also revealed the deregulation of cell cycle signals (Supplementary figure 1b, Supplementary table 3).

Because of the similarity with SCCE in pathology or genomic mutational spectrum,^12^ we also performed GSEA on SCLC and ESCC with published data on GEO database^13^ to further examine their difference on expressive level (Figure 1f, Supplementary table 4). Interestingly, immune‐associated pathways are all downregulated in SCLC while virtually upregulated in ESCC. Despite no significant enrichment, enrichment scores of immune‐associated pathways in SCCE were between those in ESCC and SCLC and closer to SCLC, which posed a requirement for further examination of immune microenvironment of SCCE. Moreover, an extended PPI network was constructed with the differentially expressed genes in SCCE (Supplementary figure 2a), suggesting a strong correlation between the deregulated genes. KEGG enrichment analysis based on the network indicated that pathways such as pathways in cancer, HTLV‐1 infection, the cell cycle, oocyte meiosis and the Wnt signalling pathway were profoundly deregulated at the transcriptome level in SCCE (Figure 1g, Supplementary table 5). Selected PPI network concerning Wnt signal also revealed the deregulating cluster of Wnt signal‐associated genes (Figure 1h, Supplementary figure 2b), in which *WNT5B*, *WNT2B* and *SFRP5* were the hubs.

### Immune microenvironment analysis of SCCE

**Figure 2:**
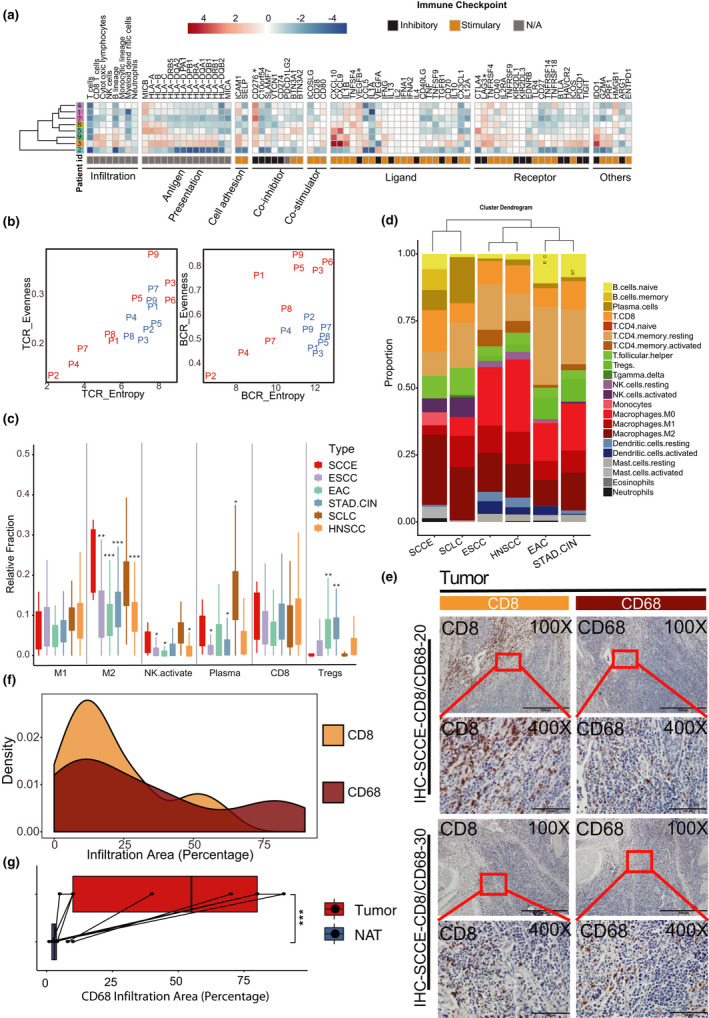
Immune microenvironment analysis of SCCE. **(a)** Hierarchical clustering and heatmap of the patients according to changes (dividing tumors by NATs) in leucocyte infiltration (left) and expression of immunomodulators (right) (red means upregulated while blue means downregulated in SCCE tissues against NATs); genes significantly upregulated in SCCE tissues against NATs are labelled with *. **(b)** Entropy and evenness of TCR (left) and BCR (right) in each sample (red, tumor tissues; blue, NATs) (NAT of patient 6 had been removed from the analysis). **(c)** Relative fraction of different types of tumor‐infiltrated leucocytes (the types of leucocytes were chosen according to their function and varying degree in different types of cancer, and the remained were shown in Supplementary figure 2d) in ESCC, EAC, STAD‐CIN, SCLC and HNSCC and their comparison with those in SCCE (Wilcoxon rank sum test with Bonferroni correction. *P‐value < 0.05; **P‐value < 0.01; ***P‐value < 0.001). **(d)** Hierarchical clustering of SCCE (*n* = 9), ESCC (*n* = 82), EAC (*n* = 80), STAD‐CIN (*n* = 207), SCLC (*n* = 81) and HNSCC (*n* = 491) by global infiltration profile with median relative fraction of 22 leucocyte types. **(e)** CD8 and CD68 IHC of SCCE tumor tissues in 2 patients. **(f)** Density distribution curve for infiltration percentage of CD8^+^ T cell (*n* = 32) and macrophage (*n* = 28). **(g)** Comparison for infiltration percentage of macrophage between SCCE tumor tissues (*n* = 7) and NATs (*n* = 7).

GSEA and KEGG analysis showed no enrichment of the immune pathways in SCCE, which might be due to the heterogeneity of tumors and the relatively low abundance of infiltrated immune cells. We employed the MCP‐counter method^14^ to obtain the absolute quantity of each type of immune cell and calculated the fold changes in immune cells from each tumor sample compared to the corresponding NAT (Figure 2a, Supplementary table 6). Most patients (6/8) showed modest infiltration of virtually all types of leucocytes in the tumor. However, two patients (patient 3 and patient 9, 2/8) had a higher leucocyte infiltration in tumor tissues than that in NATs, including CD8^+^ T cells, cytotoxic lymphocytes and B‐lineage cells (Supplementary figure 3). To further assess the infiltration of T and B cells, we examined T‐cell receptor/B‐cell receptor (TCR/BCR) diversity and clonality, which are denoted by entropy and evenness, for each case with MiXCR.^15^, ^16^ Remarkably, TCR/BCR entropy, indicating the abundance and diversity of T/B cells, was higher in NATs than in corresponding tumors (Figure 2b). Four tumor samples (patients 3, 5, 6 (without matching NAT) and 9) exhibited higher diversity and clonality of BCR, along with higher maximum counts of their BCR clone types (Supplementary table 7). Interestingly, three tumor samples (patients 3 and 6 (without matching NAT) and 9) showed relatively higher TCR entropy and TCR evenness, suggesting the higher diversity and clonality of TCR, suggesting an adaptive cell‐mediated immune microenvironment (Figure 2b, Supplementary table 8).

To further understand the immune microenvironment in individual cases, we examined the expression of all known immunomodulators.^17^ Certain well‐known inhibitors, such as B7‐H3 (cluster of different, *CD276*), lymphocyte‐activated gene 3 (LAG3), and vascular endothelial growth factor (VEGFB), and a novel immune suppressor, sialic acid‐binding Ig‐like lectin (Siglec‐15),^18^ were significantly upregulated in tumor tissues compared to corresponding NATs, which might suggest an immune evasion of SCCE (Figure 2a, Supplementary figure 4). Notably, fibrinogen‐like protein 1 (FGL1), a ligand of LAG3,^19^ was significantly upregulated in the tumor tissues of some patients showing modest infiltration of leucocytes (patients 4, 5, and 7), indicating that FGL1‐LAG3 signalling might be an inherent mechanism of the immune inhibition of SCCE. Moreover, in patients with higher leucocyte infiltration (patients 3 and 9), some immune inhibitory receptors, including PD‐1, T‐cell immunoglobulin and mucin domain‐containing protein 3 (TIM‐3) and T‐cell immunoreceptor with Ig and ITIM domains (TIGIT), and their well‐known ligands, PD‐L1, galectin 9 (Gal‐9), and PVR cell adhesion molecule (PVR), were also specifically upregulated in tumor tissues compared with NATs to varying degrees (Figure 2a, Supplementary figure 5).

To elucidate the composite features of infiltrated leucocytes in SCCE, we also used CIBERSORT to compare SCCE with cancers that exhibit similar genomic mutation profiles, including ESCC, small‐cell lung cancer (SCLC), oesophageal adenocarcinoma (EAC), chromosomal instability subtype of gastric cancer (STAD‐CIN), and head and neck squamous carcinoma (HNSCC), with RNA‐seq data from the TCGA and GEO databases (Supplementary table 9). SCCE‐infiltrated leucocytes contained more M2 macrophages and activated NK cells than other cancers except for SCLC. The fraction of plasma cells in SCCE was significantly higher than that in ESCC, STAD‐CIN and HNSCC. In contrast, fewer regulatory T cells infiltrated into SCCE than into EAC or STAD‐CIN (Figure 2c, Supplementary figure 6a). Furthermore, general infiltration into the tumors was examined by determining the median fraction of all types of leucocytes (Figure 2d). Clustering on the general infiltration spectrum suggested that the immune microenvironment of SCCE is more closely related to that of SCLC. Additionally, immunohistochemistry (IHC) assays of CD8 confirmed that 97% (29/30) of patients with SCCE had high infiltration levels of CD8^+^ T cells (Figure 2e‐g). Likewise, macrophages assayed by CD68 staining also displayed relatively high infiltration in SCCE tumor tissue compared with SCCE NATs (Figure 2e‐g, Supplementary figure 7a, b).

### Activated inflammation in the NAT of SCCE

**Figure 3:**
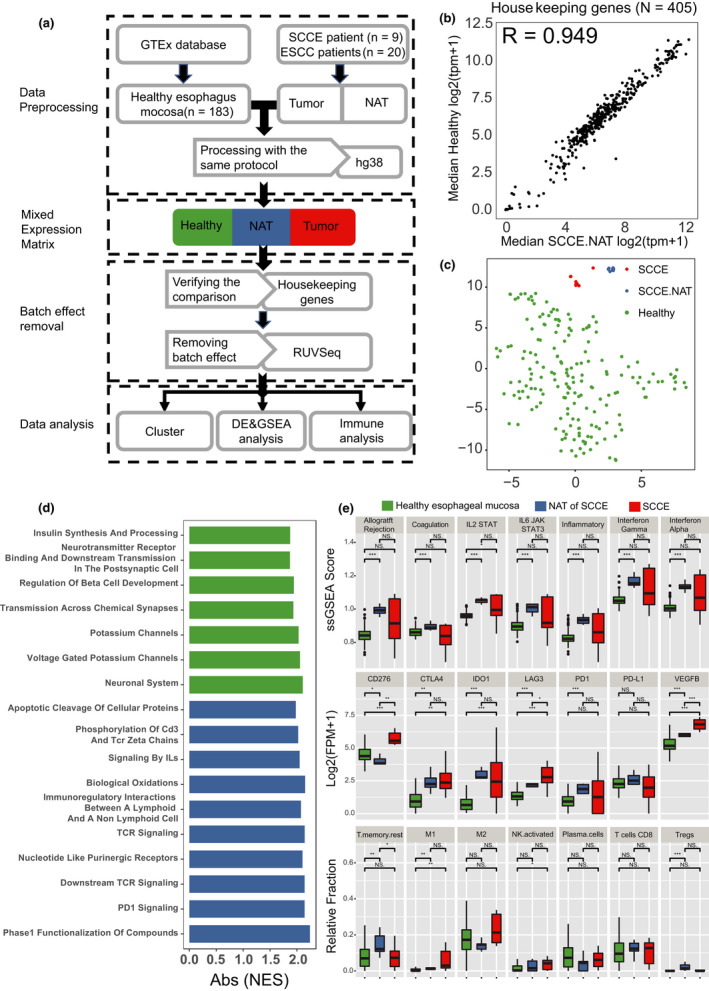
Activated inflammation in NAT of SCCE.** (a)** Process design for NAT analysis. From GTEx, we collected 183 RNA‐seq raw samples of healthy oesophageal mucosa. We performed identical processing of all samples using hg38 as reference genome and validated the data are coherent. Then, we utilised several techniques to characterise differences between healthy tissue, NAT and tumor tissue, especially in immune phenotypes. **(b)** Log_2_ expression levels of 405 housekeeping genes in healthy oesophageal mucosa tissues and NATs of SCCE (the size of the point represents the standard deviation (SD) in NAT, and the colour represents SD in healthy). **(c)** t‐SNE plots for healthy oesophageal mucosa tissues, NATs and SCCE tissues with top1000 genes ranked by median absolute deviation (MAD) of expression level across the three tissue types. **(d)** Gene set enrichment analysis of transcriptome for NATs of SCCE versus healthy oesophageal mucosa tissues (green, activating in NAT against healthy tissue; blue, suppressive in NAT against healthy tissue). **(e)** Comparison of immune phenotype including immune‐associated pathways, several suppressive immunomodulators and infiltrating leucocytes across healthy oesophageal mucosa tissues (*n* = 183), NATs (*n* = 8) and SCCE tissues (*n* = 9) (Wilcoxon rank sum test with Bonferroni correction. *P‐value < 0.05; **P‐value < 0.01; ***P‐value < 0.001).

A previous study showed that NATs differed from healthy normal tissues, with elicited inflammation.^20^ In SCCE, NATs contained more B and T cells than corresponding tumors (Figure 2b). We speculated that the NATs of SCCE have already obtained an abnormal state when comparing to the healthy oesophageal mucosa. Therefore, we took advantage of the RNA‐seq data of 183 healthy oesophageal mucosa samples from the GTEx^21^ database and unpublished RNA‐seq data of 20 paired samples of ESCC. First, all the data were preprocessed with a pipeline reported previously^20^ to remove the batch effects, allowing rigorous comparison (Figure 3a), which was shown by the expression correlation of housekeeping genes, RLE plots and PCA diagrams (Figure 3b, Supplementary figure 8a–e). Dimensionality reduction clustering of healthy tissues, NATs and tumor tissues indicated that the NATs of SCCE differed from either healthy tissues or tumor tissues at the transcriptome level (Figure 3c, Supplementary figure 10). Notably, by including ESCC data into the clustering analysis, we found that the NATs of ESCC were also different from those of healthy tissues. Moreover, SCCE, ESCC, the NATs of SCCE, the NATs of ESCC, and healthy tissues were distinct from each other at the transcriptome level (Supplementary figure 9a–c). Next, we performed differential expression analysis and GSEA to compare healthy tissues and NATs. We found that immune‐associated pathways, such as signalling by ILs, TCR signalling and downstream TCR signalling, were significantly upregulated in NATs compared with healthy tissues (Figure 3d, Supplementary table 11). Furthermore, immunomodulators, infiltrated leucocytes and immune pathways were also examined to understand the immune microenvironment of NAT in SCCE. Remarkably, all immune pathways were significantly more activated in the NATs of SCCE than in healthy oesophageal mucosa (Figure 3e). Moreover, the compositions of infiltrated leucocytes were different between healthy tissues, NATs and tumor tissues (Figure 3e, Supplementary table 12). Notably, both M1 and M2 macrophages showed higher abundance in the tumor tissues of SCCE, further suggesting a crucial role of macrophages in the tumor microenvironment of SCCE. We also found that *CTLA4* and *IDO1*, two well‐known immune checkpoints, were significantly higher in both tumor tissues and NATs than in healthy tissues, while the expression levels of *VEGFB* and *LAG3* increased progressively in healthy tissues, NATs and tumor tissues. It is noteworthy that *CD276* (B7‐H3) was the most abundant in tumors but the least abundant in NATs, which further supports its function in immune evasion in SCCE.

### Prediction of immune therapy responses based on SCCE biomarkers

**Figure 4:**
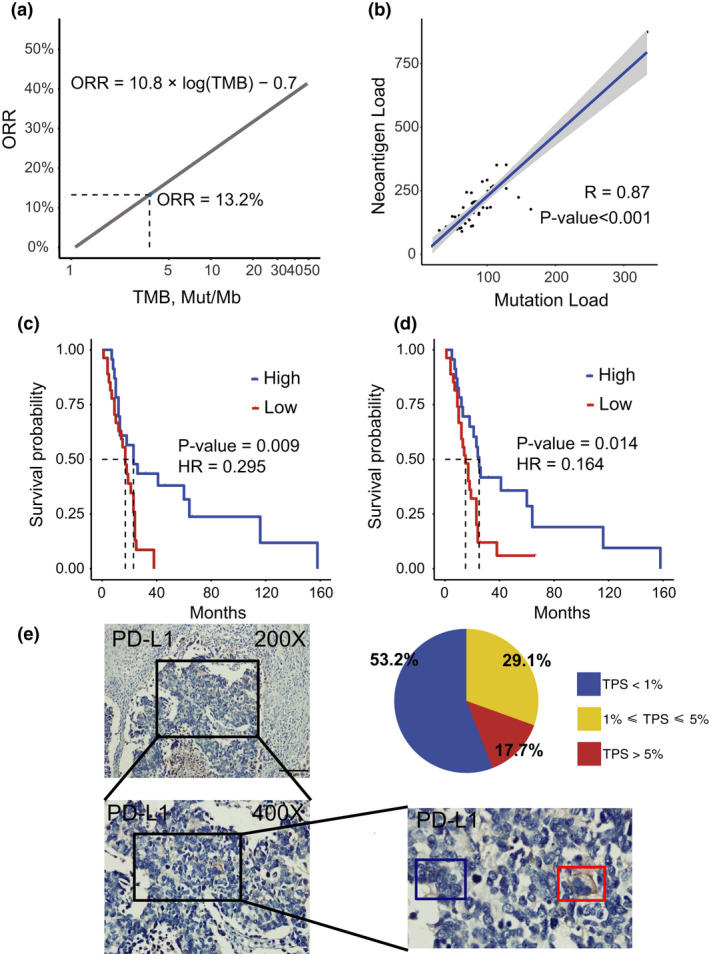
Prediction of immune therapy responses based on SCCE biomarkers. **(a)** By using linear regression model (ORR = 10.8 * log (TMB)‐0.7) published in Yarchoan *et al*.,^24^ predicted objective response rate of ICB monotherapy (anti‐PD‐1, or anti‐PD‐L1) is 13%. **(b)** Spearman correlation between mutation load and neoantigen load in 55 SCCE samples with WES. **(c)** Kaplan–Meier curves of overall survival in patients with high mutation burden (*n* = 23, more than median) and low mutation burden (*n* = 22, less than median) (log‐rank test and Cox proportional hazards model). **(d)** Kaplan–Meier curves of overall survival in patients with high neoantigen burden (*n* = 23, more than median) and low neoantigen burden (*n* = 22, less than median) (log‐rank test and Cox proportional hazards model). **(e)** PD‐L1 staining for a SCCE specimen in 200X amplification (upper left); 400X amplification for the region marked by black frame in the upper left plot (bottom left); and amplification for the region marked by black frame in the bottom left plot (bottom right). Tumor cells in the red frame were positively staining while tumor cells in the blue frame were negatively staining; distribution of TPS score for PD‐L1 staining in SCCE specimen (bottom right).

Several biomarkers, including tumor mutation burden (TMB), PD‐L1 expression, microsatellite instability and tumor neoantigen‐related mutation burden (TNB), could effectively predict the overall response of treatments with anti‐PD‐1 antibodies in certain cancers.^22^ We sought to examine whether such biomarkers exhibit the predictive value of treatments with immune checkpoint inhibitors for SCCE. We took advantage of 55 paired whole‐exome sequencing data previously published by our group^12^ and 62 SCCE samples with IHC staining retrospectively collected from 5 cancer centres. We used the MANTIS pipeline^23^ to analyse microsatellite instability and showed that all patients with SCCE (55/55) exhibited MSS (microsatellite stability). We also analysed TMB by including the synonymous mutations, and the TMB of SCCE was 3.64 [median, 95% CI, 3.18‐4.55], showing a relatively low TMB across all other tested cancer types. We then employed a TMB‐ORR linear formula^24^ to predict the response rate of SCCE for anti‐PD‐1 treatments. The predicted ORR of SCCE was 13.2% (95% CI, 11.8 ‐ 15.7, Figure 4a), with the input of the median TMB = 3.64 (95% CI, 3.18–4.55) adjusted by panel genes from Foundation One^25^ (Figure 4a). To strengthen the prediction, we also analysed neoantigen loads based on the exome data and explored their correlation with TMB in SCCE (Supplementary table 13). As expected, a strong correlation was observed between neoantigen loads and mutation loads (Figure 4b). Moreover, the analysis of previously published survival data^12^ revealed that SCCE patients with a higher mutation burden (*P*‐value = 0.009, HR = 0.295) or neoantigen burden (*P*‐value = 0.04, HR = 0.195) have a better long‐term prognosis (Figure  4c, d, Supplementary figure 11a, b).

To evaluate PD‐L1 expression in SCCE, we performed IHC staining on 62 SCCE samples retrospectively collected. Except for three specimens without tumor cell, tumor positive score (TPS) no less than 1% was detected in 44.1% of SCCE patients and TPS no less than 5% was detected in 13.6%. With the cut‐off of 1% in several clinical trials using DAKO method,^26^ 44.1% (26/59) of SCCE patients had positive PD‐L1 expression according to the cut‐off (Figure 4e; Supplementary table 14). Like most cancer types, no significant correlation was detected between TMB and PD‐L1 expression (Supplementary figure 11d–f). In addition, no significant correlation was found between CD8 T‐cell infiltration and the TMB or PD‐L1 expression, which might be due to the limited sample size (Supplementary figure 12).

## Discussion

To develop effective treatments for SCCE, a comprehensive understanding of its molecular features and immune microenvironment is urgently required. To our knowledge, this is the first study to systematically examine SCCE at the transcriptome level. Combined with our previously studied genomic profiles of SCCE, we found that genes such as *CCNE1*, *PTEN* and *PIK3R1* and pathways such as the cell cycle pathway, the P53 pathway and the Wnt signalling pathway are deregulated at both the genome and transcriptome levels, suggesting their crucial roles in the initiation and progression of SCCE. All these molecular portraits of SCCE have great potential as therapeutic targets. Besides, like SCLC, SCCE presented a trend of downregulation of immune‐associated pathways, which might be one of the factors concerning the poor prognosis of small‐cell cancers. Determining the underlying mechanism of the suppressive antitumor immunity might help to develop precise immunotherapy strategies for this deadly cancer.

Immunotherapy, especially immune checkpoint inhibitors, has achieved great success in many solid tumors, including SCLC^27^ and ESCC,^28^ both of which share similarities to SCCE.^12^ Thus, we explored the potential efficacy of immunotherapy on SCCE. We first examined the immune microenvironment of SCCE to identify suitable targets and to predict the response of immunotherapy. Previous studies^10^ have established the ‘TIME’ classification method to select patients who are more likely to respond to immunotherapy. These patients should exhibit high T‐cell infiltration and PD‐L1 expression in the tumor microenvironment. Here, we extended the definition by including B cells and immune‐stimulatory factors to reflect the immune activation state and several immune suppressive signals to explain the immune evasion of SCCE. Accordingly, with the abundant infiltration of leucocytes, T‐/B‐cell diversity, and the expression of immune‐stimulatory factors, patient 3 and patient 9 presented adaptive immune reaction. Additionally, several immune checkpoint signals, including PD‐1/PD‐L1, TIM3/Gal9 and TIGIT/PVR, were upregulated in these two patients, indicating adaptive immune resistance occurred in these two patients, which suggested that immune checkpoint inhibitors might be an effective therapeutic strategy for certain SCCE individuals. Our results also indicate that because of the complicated immune microenvironment and the expression of multiple checkpoints, pretreatment assessments and combination therapy are critical strategies to apply immunotherapy to SCCE.

However, except for patients 3 and 9, other SCCE samples showed less leucocyte infiltration, BCR/TCR diversity and lower expression of immune‐stimulatory factors in tumor tissues, presenting immunological ignorance. By profiling the immune modulators, we found that some suppressors, including B7‐H3, LAG3, VEGFB and Siglec‐15, are significantly upregulated in tumor tissues, which may contribute to the relatively suppressive immune microenvironment in SCCE. B7‐H3, encoded by CD276 gene, is a limited expressive protein in normal tissue, but aberrantly expressed in a high proportion of human malignancies.^13^ With antitumor activity against solid tumor shown by B7‐H3‐specific monoclonal antibodies (mAbs),^29^ antibody‐drug conjugates^30^ and B7‐H3‐targeting CAR‐T cells,^31^ B7‐H3 becomes a promising target for cancer immunotherapy and serves as a potential immunotherapy target for SCCE. LAG‐3 is mainly expressed on activated T and NK cells and acts as an inhibitory receptor similar to PD‐1 and CTLA‐4,^32^ which were the third inhibitory receptors to be targeted in the clinic. It is noteworthy that FGL1,^19^ a novel ligand of LAG‐3, is upregulated in patients with less leucocyte infiltration and lower PD‐1 signals, indicating that FGL1‐LAG3 might be another significant immunotherapy target for SCCE irresponsive to PD‐1/PD‐L1 blockade.

To further compare the composition of the infiltrating leucocytes of SCCE and other tumors, we integrated the transcriptomic data of ESCC, EAC, the CIN type of STAD, HNSCC, and SCLC from the TCGA and GEO databases into the analysis. M2 macrophages, the most common tumor‐associated macrophages,^33^ are the major immunoregulatory leucocytes in tumors, and they not only inhibit cytotoxic T‐cell responses in the tumor microenvironment^34^ but also propel T cells from the tumor microenvironment.^35^ Thus, the high abundance of M2 macrophages in SCCE may be one of the main reasons for the relatively low infiltration of leucocytes. Another inhibitory leucocyte is the regulatory T cell, which demonstrates low infiltration in both SCCE and SCLC, suggesting that M2 macrophages may serve as the main immune suppressive cells in SCCE and that targeting M2 macrophage^36^ may be an effective way to reverse the relatively low infiltration of leucocytes in tumor tissues.

We found that the NATs of both SCCE and ESCC are different from cancer‐free oesophageal mucosa at the transcriptome level. The NATs of several cancer types are ‘abnormal’ compared to normal tissue from healthy controls,^20^ but little is known about NATs in oesophageal cancer. Compared with healthy tissues, immune‐associated pathways are upregulated in the NATs of SCCE, implying a relatively activated immune microenvironment. Interestingly, the activation status of immune‐associated pathways in tumor tissues exhibits very large diversity, but virtually all of the pathways are relatively downregulated compared to that in corresponding NATs. Notably, the relative abundance of M2 macrophages was the lowest in NATs but the highest in tumor tissues, further indicating their suppressive roles in antitumor immunity. Instead of focusing on the immune status of tumors themselves, targeting the immune suppression of NATs might be more promising to reverse the ‘cold’ tumors.

Molecular biomarkers, such as microsatellite status, tumor mutation burden (TMB) and tumor neoantigen, have proven to have predictive value for the efficacy of immune checkpoint blockade. For instance, TMB has already been proven to be associated with efficacy of immunotherapy in melanoma, NSCLC, urinary cancer and SCLC.^37^ A previous study^24^ also revealed the positive relation between overall response rate (ORR) of ICI treatment and median TMB of a specific cancer type, and further formed a linear model. Based on 55 paired whole‐exome sequencing data, SCCE seems to have relatively stable microsatellites and a low tumor mutation burden. Despite this, after adjusting panel genes from Foundation One, the predicted ORR generated by the reported linear model still reached 13.3%. A strong correlation between somatic mutation burden and neoantigen burden also supports the prediction. Besides, both TMB and TNB were positively associated with prognosis of SCCE patients not receiving immunotherapy, suggesting that higher immunogenicity was related to better prognosis, which were probably driven by antitumor immunity. A similar phenomenon also occurs in some immunotherapy‐sensitive cancer types, such as melanoma^38^ and NSCLC.^39^, ^40^ PD‐L1 expression evaluated by IHC is another dependable biomarker in immunotherapy across several types including cancer, such as melanoma, NSCLC and HNSCC.^26^ Given that in immune microenvironment examination, high expression of PD‐1/PD‐L1 seems to be associated with adaptive immune resistance to a large extent in SCCE, we assumed that PD‐L1 expression evaluated by IHC might also serve as a potential biomarker for immunotherapy in SCCE. With the DAKO method and suggested cut‐off, our investigation revealed that considerable SCCEs were detected as having PD‐L1‐positive status. Therefore, we speculated that probably some of SCCE patients would gain benefit from immunotherapy.

However, our findings on the immune microenvironment of SCCE still remain to be validated with a larger sample size. Besides, the correlation between the deregulated immune microenvironment and intrinsic features of SCCE, such as TMB and the deregulated tumor‐associated pathways, also needs to be explored in future research, which will be helpful for elucidating the underlying mechanism and developing optimal immunotherapeutic strategies for the deadly cancer.

## Conclusions

As the first study for reporting the transcriptomic alteration of small‐cell carcinoma of the oesophagus, our results suggest that the tumor tissue of SCCE is in a macrophage‐induced suppressive immune environment and the normal adjacent tumor presented an activating immune phenotype. The examination of several ICI predictors provides a rationale for use of ICB treatment in patients with SCCE. Therefore, findings from this study provide evidence for the upcoming clinical trial design or development of immune therapy on this deadly disease.

## Methods

### RNA isolation and sequencing

Tumor purity was assessed before RNA sequencing, and only tumor samples with no less than 70% tumor tissue and normal samples without tumor cell were qualified for the sequencing. Total RNA was isolated from tissues and cells using TRIzol reagent (Life Technology, Carlsbad, USA). The isolated RNA was analysed for appropriate quantity and quality by determining the RNA integrity number (RIN) with an Agilent 2100 Bioanalyzer (Agilent Technologies, CA USA) for analysis and estimated at A260/280 nm (SmartSpec; Bio‐Rad, CA, USA) for verification. Briefly, RNA was fragmented, first‐ and second‐strand cDNA were synthesised, the double cDNA strand underwent end pair, 3’ end adenylation and adapter ligation, the second cDNA strand was digested, and libraries were amplified and purified. Lastly, the library was loaded into one lane of the Illumina HiSeq 4000 platform for sequencing.

### Transcriptome profile

The raw reads were aligned to the UCSC hg38 reference gene using STAR^41^ and assigned to genes by RSEM.^42^ Gene expression is represented by transcripts per million (TPM). Differentially expressed genes were called by the DESeq2 package. Only genes with at least 1 read in half of the samples were included for the analysis. A gene was considered differentially expressed if (i) the adjusted *P*‐value was < 0.05 and (ii) there was a >2‐fold expression change. Principal component analysis (PCA) was performed with *plotPCA* function from the DESeq2^43^ package. GSEA within the Kyoto Encyclopedia of Genes and Genomes (KEGG) database, Gene Ontology (GO) and hallmark gene sets were conducted by employing WebGestalt (http://www.webgestalt.org/) with ranked gene lists (by fold change). Enrichment score was considered as informative only if the adjusted *P* value (also marked as FDR, False Discovery Rate) < 0.05. An expanded PPI network was created with the top 500 upregulated genes and the top 500 downregulated genes by using NetworkAnalyst,^44^ and KEGG enrichment on the network was also performed on the website.

### Immune microenvironment examination

The absolute infiltrating abundance of leucocytes was calculated by the MCP‐counter method (version 1.1).^14^ The expression levels of immunomodulators are denoted in TPM. The identification of TCR CDR3 sequences from T cells present in the sequenced tumor sections was examined by MiXCR from RNA‐seq data.^16^ The entropy of the receptor repertoire was calculated by the tcR package,^45^ while the evenness of the receptor repertoire was denoted by the Gini coefficient calculated with the ineq package in R. The relative fractions of 22 types of tumor‐infiltrating leucocytes in the multi‐cancer comparison were calculated by CIBERSORT.^46^, ^47^

### Exploration of the nature of NAT

#### Raw data collection and processing

We obtained raw read files of healthy oesophagus mucosa tissue from the GTEx database (GTEx dbGaP accession phs000424.v7. p2.c1, 18 September, 2018). Raw read files of SCLC were obtained from GEO database (GSE60052). The raw reads were analysed by using the identical pipeline to SCCE data set.

#### Batch effects removal

In the comparison of RNA‐seq data from healthy oesophagus mucosa, normal tissue adjacent to the tumor (NAT) and SCCE tissue, batch effects and differences in sample preparation can have a substantial influence on outcomes. Thus, we applied raw data from the GTEx database and preprocessed the data in the identical workflow that was used to preprocess the SCCE and normal tissue data. In addition, we employed the *RUVg* method in the RUVseq package, which can remove noisy expression from RNA‐seq data using a negative gene set that has constant covariates.^48^ The negative gene set we used in the article was a list of housekeeping genes^49^ that were recommend by the developer of the method. As observed in the relative log expression (RLE) and PCA cluster with housekeeping genes, this procedure can diminish unwanted variation between data.

#### Bioinformatics analysis

Dimensionality reduction clustering by the t‐SNE algorithm^50^ when clarifying the relationship in the expression profiles between SCCE, normal tissue adjacent to SCCE and healthy oesophagus mucosa was performed using the Rtsne (version 0.15) package with the top 1000 genes ranked by median absolute deviation. GSEA within Reactome was performed with the identical GSEA method mentioned in the transcriptome profile to compare NAT and healthy tissue. The ssGSEA scores of hallmark immune pathways were calculated by the GSVA package in R.^51^ The fraction of tissue‐infiltrating leucocytes was also determined with CIBERSORT.

### Tumor foreignness assessment

MSI was evaluated using MANTIS (version 1.0.4)^52^ on whole‐exon sequencing data of 55 paired SCCE samples. The TMB of all protein‐coding genes and the selected panel from Foundation One were calculated separately. HLA class I typing of samples was performed using the OptiType tool (version 1.3).^53^ Potential neoantigenic peptides were identified using NeoPredPipe based on netMHCpan (version 4.0).^54^

### Statistical analysis

The Spearman rank correlation coefficient was used to measure the relationship between two variables. The Wilcoxon rank sum test and the Wilcoxon matched‐pairs signed rank sum test with Bonferroni correction were used to compare the difference of two or more sets of quantitative data. Distributions of overall survival (OS) were described by Kaplan–Meier methods, and *P* values were calculated using a two‐sided log‐rank test. Univariate and multivariable Cox proportional hazards models were used to predict factors that influence the outcome. All *P*‐values were two‐sided, and *P*‐values less than 0.05 were considered statistically significant.

### Immunohistochemical assay

Tumor samples were evaluated for CD8 (Beijing Zhongshan Jinqiao Biotechnology [ZSJQB], Co., Ltd., Beijing, ZA‐0508‐6.0), CD68 (ZSJQB, Co., Ltd., Beijing, ZM‐0060‐6.0) and PD‐L1 (Dako, M365329) expression through immunohistochemistry (IHC) staining by certified pathologists. The numbers of CD8^+^ lymphocytes and CD68^+^ macrophages were manually counted under a high‐power field (400×). PD‐L1‐positive status was defined as the presence of membrane staining of any intensity in ≥1% of tumor cells or the presence of PD‐L1 staining of any intensity in tumor‐infiltrating immune cells covering ≥1% of the tumor area occupied by tumor cells associated with the intratumoral and contiguous peritumoral stromal.

## Conflicts of interests

The authors declare no conflict of interest.

## Author Contribution

**Qi Zhao:** Conceptualization; Data curation; Investigation; Methodology; Resources; Software; Supervision; Visualization; Writing‐original draft; Writing‐review & editing. **Yan‐Xing Chen:** Data curation; Investigation; Methodology; Software; Visualization; Writing‐original draft; Writing‐review & editing. **Qi‐Nian Wu:** Data curation; Investigation; Methodology; Resources; Validation; Writing‐original draft; Writing‐review & editing. **Chao Zhang:** Data curation; Resources; Validation; Writing‐review & editing. **Min Liu:** Data curation; Resources. **Ying‐Nan Wang:** Data curation; Resources; Validation. **Yan‐Fen Feng:** Resources; Validation. **Jia‐Jia Hu:** Formal analysis; Resources; Validation. **Jian‐Hua Fu:** Investigation; Resources; Writing‐review & editing. **Hong Yang:** Investigation; Resources; Validation; Writing‐review & editing. **JingJing Qi:** Formal analysis; Software; Writing‐review & editing. **Zi‐Xian Wang:** Data curation; Formal analysis; Investigation. **Yun‐Xin Lu:** Formal analysis; Validation; Writing‐review & editing. **Hui Sheng:** Data curation; Validation; Visualization. **Ze‐Xian Liu:** Formal analysis; Investigation; Methodology; Software. **Zhi‐Xiang Zuo:** Investigation; Methodology; Software; Visualization. **Jian Zheng:** Investigation; Methodology; Visualization; Writing‐review & editing. **Jing‐Ping Yun:** Data curation; Investigation; Resources. **Jin‐Xin Bei:** Conceptualization; Investigation; Methodology. **Wei‐Hua Jia:** Conceptualization; Investigation; Methodology. **Dong‐Xin Lin:** Conceptualization; Investigation; Methodology. **Rui‐Hua Xu:** Conceptualization; Funding acquisition; Investigation; Supervision; Writing‐review & editing. **Feng Wang:** Conceptualization; Funding acquisition; Investigation; Project administration; Writing‐original draft; Writing‐review & editing.

## Ethics approval and consent to participate

Permission for the study was obtained from the Ethics Committee of Sun Yat‐Sen University Cancer Center. All human materials were collected after approval of the institutional review board with protocol number YP2013‐07‐04, ‘Genetic profiling of small cell carcinoma of esophagus’.

## Data Availability

The data sets used and analysed during the current study are available from the corresponding author on reasonable request.

## References

[1] Hudson E , Powell J , Mukherjee S *et al* Small cell oesophageal carcinoma: an institutional experience and review of the literature. Br J Cancer 2007; 96: 708–711. 17299393 10.1038/sj.bjc.6603611 PMC2360086

[2] Chen WW , Wang F , Chen S *et al* Detailed analysis of prognostic factors in primary esophageal small cell carcinoma. Ann Thorac Surg 2014; 97: 1975–1981. 24726599 10.1016/j.athoracsur.2014.02.037

[3] Song Y , Wang W , Tao G , Zhu W , Zhou X , Pan P . Survival benefit of radiotherapy to patients with small cell esophagus carcinoma: an analysis of Surveillance Epidemiology and End Results (SEER) data. Oncotarget 2016; 7: 15474–15480. 26943276 10.18632/oncotarget.6764 PMC4941254

[4] Brenner B , Tang LH , Klimstra DS , Kelsen DP . Small‐cell carcinomas of the gastrointestinal tract: a review. J Clin Oncol 2004; 22: 2730–2739. 15226341 10.1200/JCO.2004.09.075

[5] Canales ES , Zarate A , Gonzales A , MacGregor C , Villalobos M , Delgado J . Occurrence of spontaneous second pregnancy after delivery of conceptions resulting from ovulation induced by various therapeutic modalities. Int J Fertil 1973; 18: 182–184. 4357284

[6] Topalian SL , Hodi FS , Brahmer JR *et al* Safety, activity, and immune correlates of anti‐PD‐1 antibody in cancer. N Engl J Med 2012; 366: 2443–2454. 22658127 10.1056/NEJMoa1200690 PMC3544539

[7] Schulze AB , Schmidt LH . PD‐1 targeted Immunotherapy as first‐line therapy for advanced non‐small‐cell lung cancer patients. J Thorac Dis 2017; 9: e384–e386. 28523184 10.21037/jtd.2017.03.118 PMC5418249

[8] Motzer RJ , Tannir NM , McDermott DF *et al* Nivolumab plus Ipilimumab versus Sunitinib in Advanced Renal‐Cell Carcinoma. N Engl J Med 2018; 378: 1277–1290. 29562145 10.1056/NEJMoa1712126 PMC5972549

[9] Tang H , Qiao J , Fu YX . Immunotherapy and tumor microenvironment. Cancer Lett 2016; 370: 85–90. 26477683 10.1016/j.canlet.2015.10.009 PMC4725050

[10] Teng MW , Ngiow SF , Ribas A , Smyth MJ . Classifying Cancers Based on T‐cell Infiltration and PD‐L1. Cancer Res 2015; 75: 2139–2145. 25977340 10.1158/0008-5472.CAN-15-0255 PMC4452411

[11] Wang L , Wang S , Li W . RSeQC: quality control of RNA‐seq experiments. Bioinformatics 2012; 28: 2184–2185. 22743226 10.1093/bioinformatics/bts356

[12] Wang F , Liu DB , Zhao Q *et al* The genomic landscape of small cell carcinoma of the esophagus. Cell Res 2018; 28: 771–774. 29728688 10.1038/s41422-018-0039-1 PMC6028429

[13] Jiang L , Huang J , Higgs BW *et al* Genomic Landscape Survey Identifies SRSF1 as a Key Oncodriver in Small Cell Lung Cancer. PLoS Genet 2016; 12: e1005895. 27093186 10.1371/journal.pgen.1005895 PMC4836692

[14] Becht E , Giraldo NA , Lacroix L *et al* Estimating the population abundance of tissue‐infiltrating immune and stromal cell populations using gene expression. Genome Biol 2016; 17: 218. 27765066 10.1186/s13059-016-1070-5 PMC5073889

[15] Kirsch I , Vignali M , Robins H . T‐cell receptor profiling in cancer. Mol Oncol 2015; 9: 2063–2070. 26404496 10.1016/j.molonc.2015.09.003 PMC5528728

[16] Bolotin DA , Poslavsky S , Mitrophanov I *et al* MiXCR: software for comprehensive adaptive immunity profiling. Nat Methods 2015; 12: 380–381. 25924071 10.1038/nmeth.3364

[17] Tang J , Shalabi A , Hubbard‐Lucey VM . Comprehensive analysis of the clinical immuno‐oncology landscape. Ann Oncol 2018; 29: 84–91. 29228097 10.1093/annonc/mdx755

[18] Wang J , Sun J , Liu LN *et al* Siglec‐15 as an immune suppressor and potential target for normalization cancer immunotherapy. Nat Med 2019e; 25: 656–666. 30833750 10.1038/s41591-019-0374-x PMC7175920

[19] Wang J , Sanmamed MF , Datar I *et al* Fibrinogen‐like Protein 1 Is a Major Immune Inhibitory Ligand of LAG‐3. Cell 2019; 176: 334–347. e312. 30580966 10.1016/j.cell.2018.11.010 PMC6365968

[20] Aran D , Camarda R , Odegaard J *et al* Comprehensive analysis of normal adjacent to tumor transcriptomes. Nat Commun 2017; 8: 1077. 29057876 10.1038/s41467-017-01027-z PMC5651823

[21] Consortium GT . The Genotype‐Tissue Expression (GTEx) project. Nat Genet 2013; 45: 580–585. 23715323 10.1038/ng.2653 PMC4010069

[22] Le DT , Durham JN , Smith KN *et al* Mismatch repair deficiency predicts response of solid tumors to PD‐1 blockade. Science 2017; 357: 409–413. 28596308 10.1126/science.aan6733 PMC5576142

[23] Bonneville R , Krook MA , Kautto EA *et al* Landscape of Microsatellite Instability Across 39 Cancer Types. JCO Precis Oncol 2017; 2017: 1–15. 10.1200/PO.17.00073 PMC5972025 29850653

[24] Yarchoan M , Hopkins A , Jaffee EM . Tumor Mutational Burden and Response Rate to PD‐1 Inhibition. N Engl J Med 2017; 377: 2500–2501. 29262275 10.1056/NEJMc1713444 PMC6549688

[25] Chalmers ZR , Connelly CF , Fabrizio D *et al* Analysis of 100,000 human cancer genomes reveals the landscape of tumor mutational burden. Genome Med 2017; 9: 34. 28420421 10.1186/s13073-017-0424-2 PMC5395719

[26] Diggs LP , Hsueh EC . Utility of PD‐L1 immunohistochemistry assays for predicting PD‐1/PD‐L1 inhibitor response. Biomark Res 2017; 5: 12. 28331612 10.1186/s40364-017-0093-8 PMC5353958

[27] Cooper MR , Alrajhi AM , Durand CR . Role of immune checkpoint inhibitors in small cell lung cancer. Am J Ther 2018; 25: e349–e356. 29722737 10.1097/MJT.0000000000000686

[28] Kojima T , Doi T . Immunotherapy for Esophageal Squamous Cell Carcinoma. Curr Oncol Rep 2017; 19: 33. 28361224 10.1007/s11912-017-0590-9 PMC5374168

[29] Fauci JM , Sabbatino F , Wang Y *et al* Monoclonal antibody‐based immunotherapy of ovarian cancer: targeting ovarian cancer cells with the B7‐H3‐specific mAb 376.96. Gynecol Oncol 2014; 132: 203–210. 24216048 10.1016/j.ygyno.2013.10.038

[30] Kasten BB , Arend RC , Katre AA *et al* B7‐H3‐targeted (212)Pb radioimmunotherapy of ovarian cancer in preclinical models. Nucl Med Biol 2017; 47: 23–30. 28104527 10.1016/j.nucmedbio.2017.01.003 PMC5340614

[31] Du H , Hirabayashi K , Ahn S *et al* Antitumor responses in the absence of toxicity in solid tumors by targeting B7–H3 via chimeric antigen receptor T cells. Cancer Cell 2019; 35: 221–237.e228. 30753824 10.1016/j.ccell.2019.01.002 PMC6645919

[32] Long L , Zhang X , Chen F *et al* The promising immune checkpoint LAG‐3: from tumor microenvironment to cancer immunotherapy. Genes Cancer 2018; 9: 176–189. 30603054 10.18632/genesandcancer.180 PMC6305110

[33] Grivennikov SI , Greten FR , Karin M . Immunity, inflammation, and cancer. Cell 2010; 140: 883–899. 20303878 10.1016/j.cell.2010.01.025 PMC2866629

[34] Yang L , Zhang Y . Tumor‐associated macrophages: from basic research to clinical application. J Hematol Oncol 2017; 10: 58. 28241846 10.1186/s13045-017-0430-2 PMC5329931

[35] Joyce JA , Fearon DT . T cell exclusion, immune privilege, and the tumor microenvironment. Science 2015; 348: 74–80. 25838376 10.1126/science.aaa6204

[36] Poh AR , Ernst M . Targeting Macrophages in Cancer: From Bench to Bedside. Front Oncol 2018; 8: 49. 29594035 10.3389/fonc.2018.00049 PMC5858529

[37] Chan TA , Yarchoan M , Jaffee E *et al* Development of tumor mutation burden as an immunotherapy biomarker: utility for the oncology clinic. Ann Oncol 2019; 30: 44–56. 30395155 10.1093/annonc/mdy495 PMC6336005

[38] Simpson D , Ferguson R , Martinez CN *et al* Mutation burden as a potential prognostic marker of melanoma progression and survival. Am Soc Clin Oncol 2017; 35: 9567.

[39] Kang J , Luo Y , Wang D *et al* Tumor mutation load: a novel independent prognostic factor in stage IIIA‐N2 Non‐small‐cell lung Cancer. Dis Markers 2019; 2019: 3837687. 31182981 10.1155/2019/3837687 PMC6515152

[40] Devarakonda S , Rotolo F , Tsao MS *et al* Tumor mutation burden as a biomarker in resected non‐small‐cell lung cancer. J Clin Oncol 2018; 36: 2995–3006. 30106638 10.1200/JCO.2018.78.1963 PMC6804865

[41] Dobin A , Davis CA , Schlesinger F *et al* STAR: ultrafast universal RNA‐seq aligner. Bioinformatics 2013; 29: 15–21. 23104886 10.1093/bioinformatics/bts635 PMC3530905

[42] Li B , Dewey CN . RSEM: accurate transcript quantification from RNA‐Seq data with or without a reference genome. BMC Bioinform 2011; 12: 323. 10.1186/1471-2105-12-323 PMC3163565 21816040

[43] Love MI , Huber W , Anders S . Moderated estimation of fold change and dispersion for RNA‐seq data with DESeq2. Genome Biol 2014; 15: 550. 25516281 10.1186/s13059-014-0550-8 PMC4302049

[44] Xia J , Benner MJ , Hancock RE . NetworkAnalyst–integrative approaches for protein‐protein interaction network analysis and visual exploration. Nucleic Acids Res 2014; 42: W167–W174. 24861621 10.1093/nar/gku443 PMC4086107

[45] Nazarov VI , Pogorelyy MV , Komech EA *et al* tcR: an R package for T cell receptor repertoire advanced data analysis. BMC Bioinform 2015; 16: 175. 10.1186/s12859-015-0613-1 PMC4445501 26017500

[46] Newman AM , Liu CL , Green MR *et al* Robust enumeration of cell subsets from tissue expression profiles. Nat Methods 2015; 12: 453–457. 25822800 10.1038/nmeth.3337 PMC4739640

[47] Gentles AJ , Newman AM , Liu CL *et al* The prognostic landscape of genes and infiltrating immune cells across human cancers. Nat Med 2015; 21: 938–945. 26193342 10.1038/nm.3909 PMC4852857

[48] Risso D , Ngai J , Speed TP , Dudoit S . Normalization of RNA‐seq data using factor analysis of control genes or samples. Nat Biotechnol 2014; 32: 896–902. 25150836 10.1038/nbt.2931 PMC4404308

[49] Eisenberg E , Levanon EY . Human housekeeping genes, revisited. Trends Genet 2013; 29: 569–574. 23810203 10.1016/j.tig.2013.05.010

[50] Lvd Maaten , Hinton G . Visualizing data using t‐SNE. J Mach Learn Res 2008; 9: 2579–2605.

[51] Foroutan M , Bhuva DD , Lyu R , Horan K , Cursons J , Davis MJ . Single sample scoring of molecular phenotypes. BMC Bioinform 2018; 19: 404. 10.1186/s12859-018-2435-4 PMC6219008 30400809

[52] Kautto EA , Bonneville R , Miya J *et al* Performance evaluation for rapid detection of pan‐cancer microsatellite instability with MANTIS. Oncotarget 2017; 8: 7452–7463. 27980218 10.18632/oncotarget.13918 PMC5352334

[53] Szolek A , Schubert B , Mohr C , Sturm M , Feldhahn M , Kohlbacher O . OptiType: precision HLA typing from next‐generation sequencing data. Bioinformatics 2014; 30: 3310–3316. 25143287 10.1093/bioinformatics/btu548 PMC4441069

[54] Jurtz V , Paul S , Andreatta M , Marcatili P , Peters B , Nielsen M . NetMHCpan‐4.0: improved peptide‐MHC class I interaction predictions integrating eluted ligand and peptide binding affinity data. J Immunol 2017; 199: 3360–3368. 28978689 10.4049/jimmunol.1700893 PMC5679736

