# Supplementary material for: Systematic analysis of the transcriptome in small‐cell carcinoma of the oesophagus reveals its immune microenvironment
Source: Clin Transl Immunology. 2020 Oct 5;9(10):e1173. doi: 10.1002/cti2.1173 (PMC7536114; doi:10.1002/cti2.1173)

Supplementary materials

Title: **Systematic analysis of the transcriptome in small-cell carcinoma of the esophagus reveals its immune microenvironment**

Qi Zhao[1,]†, Yan-Xing Chen[1,]†, Qi-Nian Wu[2,]†, Chao Zhang[2], Min Liu[1], Ying-Nan Wang[1], Yan-Fen Feng[2], Jia-Jia Hu[1], Jian-Hua Fu[1], Hong Yang[1], Jing-Jing Qi[1], Zi-Xian Wang[1], Yun-Xin Lu[1], Hui Sheng[1], Ze-Xian Liu[1], Zhi-Xiang Zuo[1], Jian Zheng[1], Jing-Ping Yun[1], Jin-Xin Bei[1], Wei-Hua Jia[1], Dong-Xin Lin[1], Rui-hua Xu[1] , Feng Wang[1,]*

1. State Key Laboratory of Oncology in South China, Collaborative Innovation Center for Cancer Medicine, Sun Yat-sen University Cancer Center, 651 E Dongfeng Road, Guangzhou, Guangdong, 510060, China
2. Department of Pathology, Sun Yat-sen University Cancer Center, State Key Laboratory of Oncology in South China, Collaborative Innovation Center for Cancer Medicine, Guangzhou, 510060, China

*To whom correspondence should be addressed.

†The authors wish it to be known that, in their opinion, the first three authors contributed equally to this work.

Supplementary tables

Supplementary table 1. Differential expression analysis between tumor tissues and their NATs by using DESeq2.

Supplementary table 2. HALLMARK GSEA for SCCE tumor tissues versus NATs

Supplementary table 3. GO enrichment analysis for differentially expressed genes in SCCE

Supplementary table 4. HALLMARK GSEA for ESCC and SCLC based on comparison between tumors and matching normal tissues

Supplementary table 5. KEGG enrichment analysis for extended network established with DEGs in SCCE

Supplementary table 6. Absolute abundance of tissue infiltrating leukocyte calculated with MCP-counter

Supplementary table 7. BCR analysis for tumor tissues and NATs of SCCE with transcriptome data

Supplementary table 8. TCR analysis for tumor tissues and NATs of SCCE with transcriptome data

Supplementary table 9. Relative abundance of tumor infiltrating leukocyte calculated with CIBERSORT in SCCE, SCLC, ESCC, EAC, CIN type of STAD (STAD.CIN) and HNSCC

Supplementary table 10. IHC assays of CD8 and CD68 for SCCE specimens

Supplementary table 11. Reactome GSEA for NATs of SCCE versus healthy tissues

Supplementary table 12. Relative abundance of tumor infiltrating leukocyte calculated with CIBERSORT in SCCE tumor tissues, SCCE NATs and healthy tissues

Supplementary table 13. Neoantigen count of 55 SCCE samples with whole exome sequencing data

Supplementary table 14. IHC assays of PD-L1 for SCCE specimens

Supplementary figures

Supplementary figure 1 Extended transcriptomic profiling of SCCE

(a) Correlation across the transcriptome of 9 paired samples. Matching normal tissues of patient6 presented an abnormal status with low correlation with other samples and was removed in the following analysis. **(b)** GO enrichment analysis for the differentially expressed genes in SCCE tissues versus NATs of SCCE.

Supplementary figure2 Extended deregulated PPI network of SCCE

(a) The whole extended PPI network with Wnt signal associated genes annotated. **(b)** Subnetwork concerning Wnt signal (cluster in the red frame was enlarged as **figure 1h**).

Supplementary figure 3 Paired comparison of infiltration leukocytes

(a) Paired comparison of infiltration leukocytes in each sample. (Blue, NATs, n=8; red, SCCE, n=8).

Supplementary figure 4 Immunomodulators upregulated in SCCE

(a) Several inhibitive immunomodulators (CD276, VEGFB, Siglec-15, LAG-3) significantly upregulated in SCCE tissues against NATs of SCCE. FGL1 was reported by Chen L et al. as a novel ligand of LAG-3, showing an upregulation in tumor tissues in some cases (Wilcoxon matched-pairs signed ranks sum test with Bonferroni correction).

Supplementary figure 5 Expression of well-known immunomodulators in SCCE

(a) Paired comparison of well-known immune checkpoints including PD-1, TIM-3 and TIGIT and their ligands CD274(PD-L1), Gal-9 and PVR. CTLA-4 and IDO1 were also included.

Supplementary figure 6 Comparison of infiltration composition for multi-cancer

(a) Relative fraction of remained types (not shown in figure2C) of tumor infiltrated leukocytes in ESCC (n=82), EAC (n=80), STAD-CIN (n=207), SCLC (n=81) and HNSCC (n=491) and their comparison with those in SCCE (Wilcox rank sum test with Bonferroni correction. *, P-value < 0.05; **, P-value < 0.01; ***, P-value < 0.001).

Supplementary figure 7 MHC staining result in SCCE tumor tissues and NATs. (a) Frequency of each IHC staining intensity level for CD8 and CD68 in SCCE samples. **(b)** CD68 IHC of SCCE NATs in 2 patients.

Supplementary figure 8 Transcriptomic comparison between NAT of SCCE and healthy tissues.

(a) Log2 expression levels of 405 housekeeping genes in healthy esophageal mucosa tissues and NATs of ESCC. **(b)** Log2 expression levels of 405 housekeeping genes in healthy esophageal mucosa tissues and ESCC tissues. **(c)** Log2 expression levels of 405 housekeeping genes in healthy esophageal mucosa tissues and SCCE. **(d)** Relative Log Expression (RLE) plot of SCCE tissues (n=9), NATs of SCCE (n=8) and healthy esophageal mucosa tissues (n=183) via batch effect removal processes. **(e)** PCA plot of SCCE tissues (n=9), NATs of SCCE (n=8) and healthy esophageal mucosa tissues (n=183) via batch effect removal processes.

Supplementary figure 9 t-SNE plots for healthy esophageal mucosa tissues (n=9),

NATs (n=8) and SCCE tissues (n=183) with different number of genes. (a) t-SNE plots for healthy esophageal mucosa tissues, NATs and SCCE tissues with top500 genes ranked by median absolute deviation (MAD) of expression level across the three tissue types. **(b)** t-SNE plots for healthy esophageal mucosa tissues, NATs and SCCE tissues with top200 genes ranked by median absolute deviation (MAD) of expression level across the three tissue types. **(c)** t-SNE plots for healthy esophageal mucosa tissues, NATs and SCCE tissues with top5000 genes ranked by median absolute deviation (MAD) of expression level across the three tissue types. **(d)** t-SNE plots for healthy esophageal mucosa tissues, NATs and SCCE tissues with top10000 genes ranked by median absolute deviation (MAD) of expression level across the three tissue types.

Supplementary figure 10 Relationship between SCCE, NATs of SCCE, ESCC, NATs of ESCC and heathy esophageal mucosa tissues.

(a) Relative Log Expression (RLE) plot of SCCE tissues, NATs of SCCE, ESCC, NATs of ESCC and healthy esophageal mucosa tissues via batch effect removal processes. **(b)** PCA plot of SCCE tissues, NATs of SCCE, ESCC, NATs of ESCC and healthy esophageal mucosa tissues via batch effect removal processes. **(c)** t-SNE plot for SCCE tissues, NATs of SCCE, ESCC, NATs of ESCC and healthy esophageal mucosa tissues.

Supplementary figure 11 Evaluation of Immunotherapy predictors in SCCE

(a) Kaplan–Meier curves of overall survival in patients with high strong binding neoantigen burden (more than median) and low strong binding neoantigen (NeoantigenSB) burden (less than median) (Log-rank test and Cox Proportional-Hazards Model). **(b)** Kaplan–Meier curves of overall survival in patients with high

weak binding neoantigen burden (more than median) and low weak binding neoantigen (NeoantigenWB) burden (less than median) (Log-rank test and Cox Proportional-Hazards Model). **(c)** Microsatellite status analysis for SCCE with MANTIS. **(d)** Venn plot for the number of overlapping samples. **(e)** Mutation Load in SCCE samples with different PD-L1 IHC status (Positive: TPS ⩾1%; Negative: TPS < 1%). **(f)** Correlation between PD-L1-IHC CPS score and Mutation Load in SCCE samples.

Supplementary figure 12 Correlation between immunotherapy predictors in SCCE

(a) Mutation load in SCCE samples with different CD8 IHC staining intensities. **(b)** Correlation between the percentage of CD8 infiltration and the mutation load in SCCE samples. **(c)** PD-L1 TPS in SCCE samples with different CD8 IHC staining intensities. **(d)** Correlation between the percentage of CD8 infiltration and the PD-L1 TPS in SCCE samples.

Supplementary figure 1

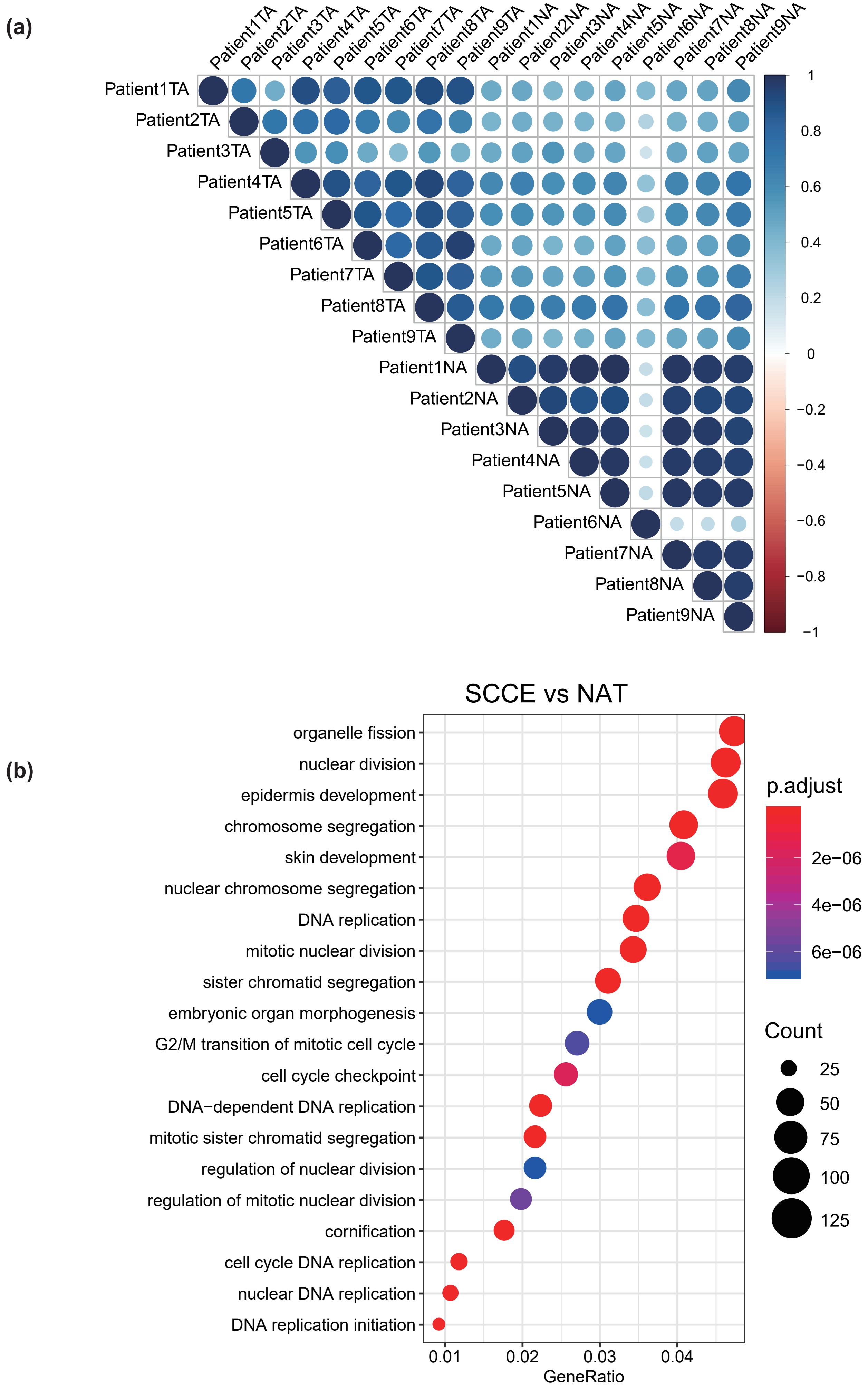

Supplementary figure 2

(a)

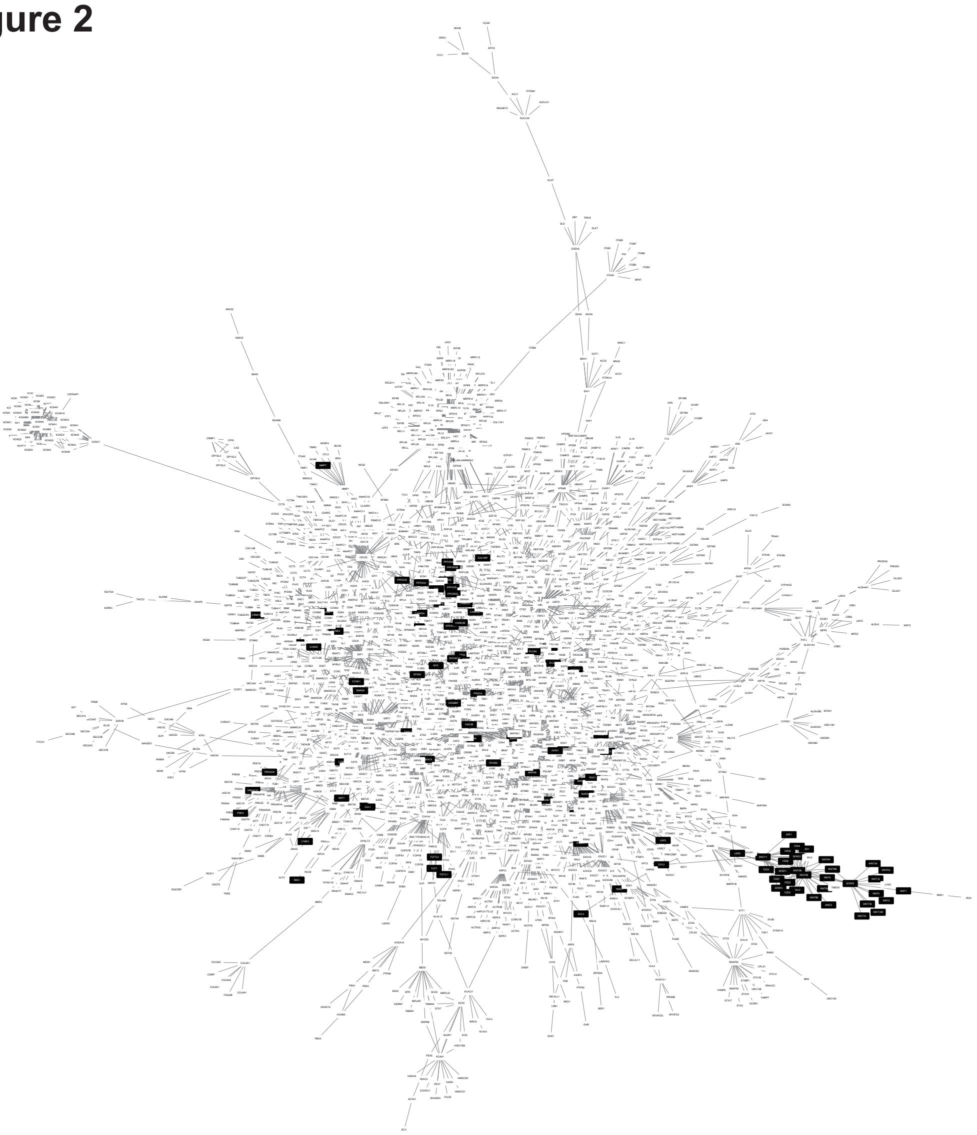

(b)

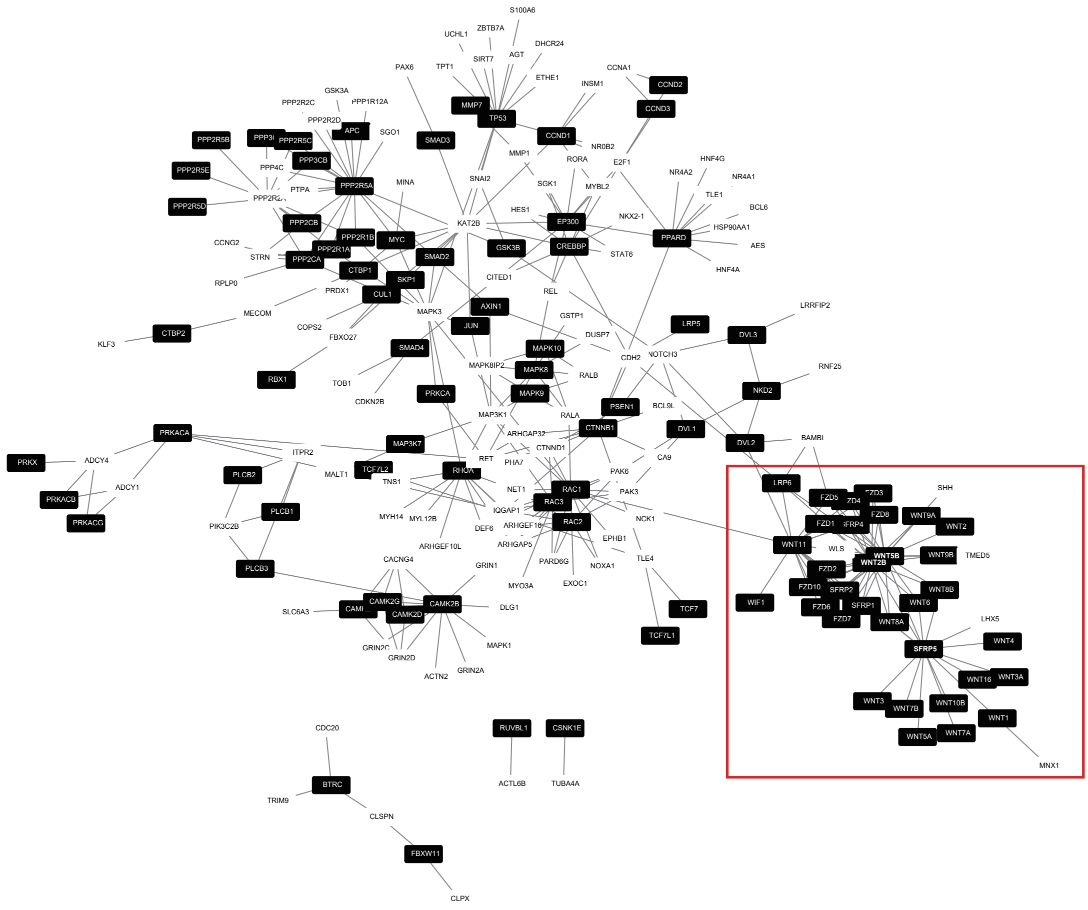

Supplementary figure 3

(a)

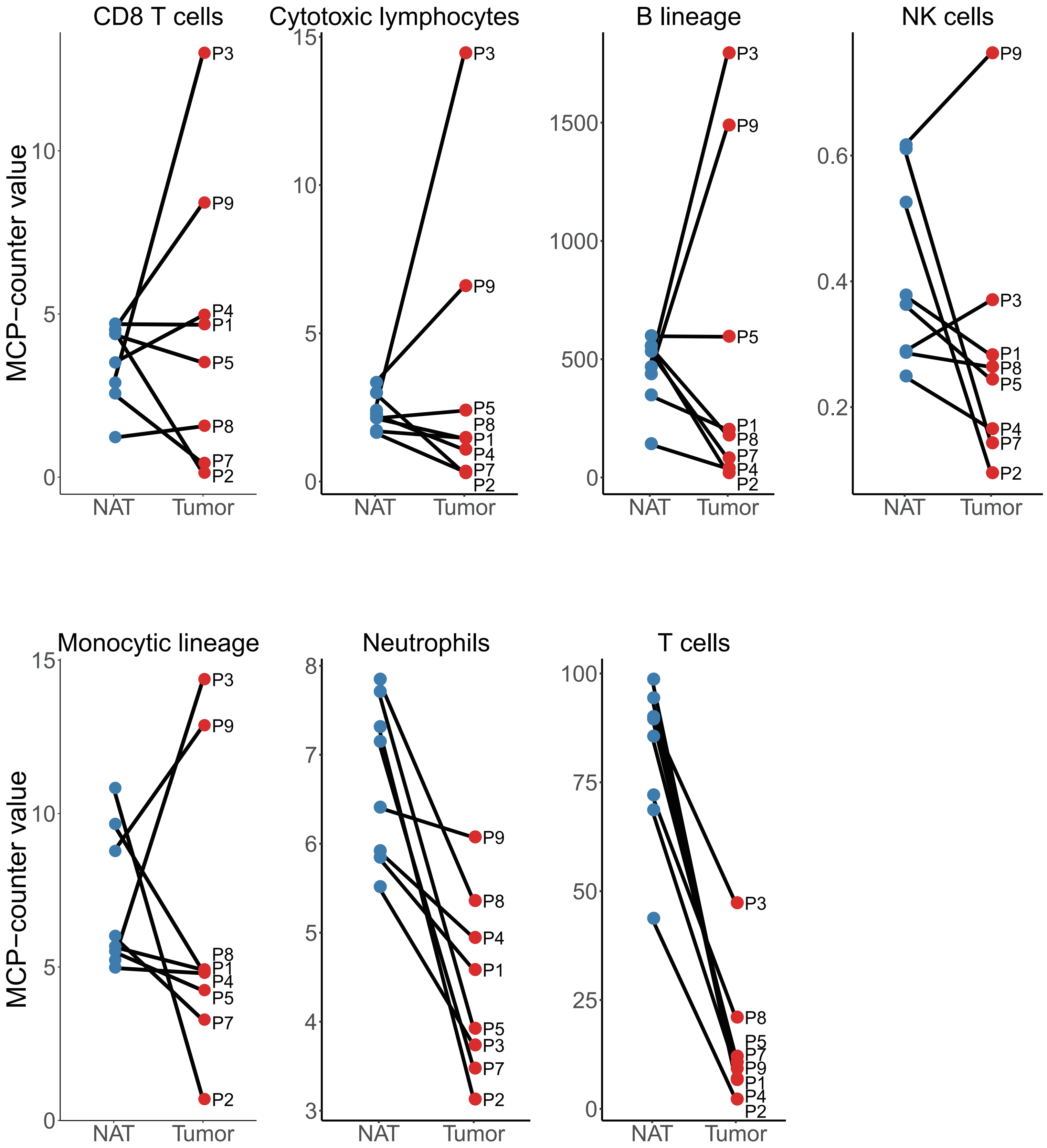

Supplementary figure 4

(a)

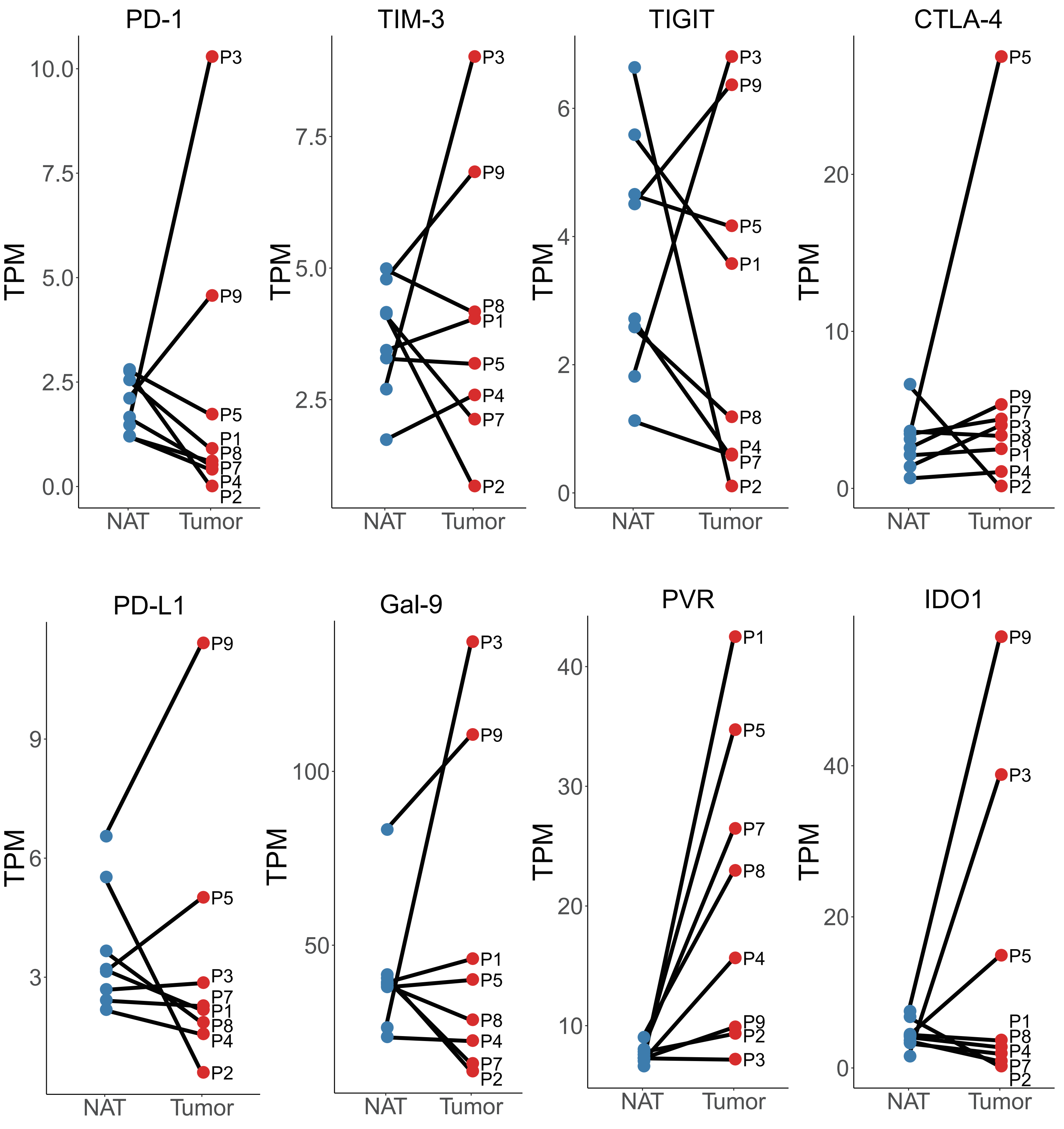

Supplementary figure 5

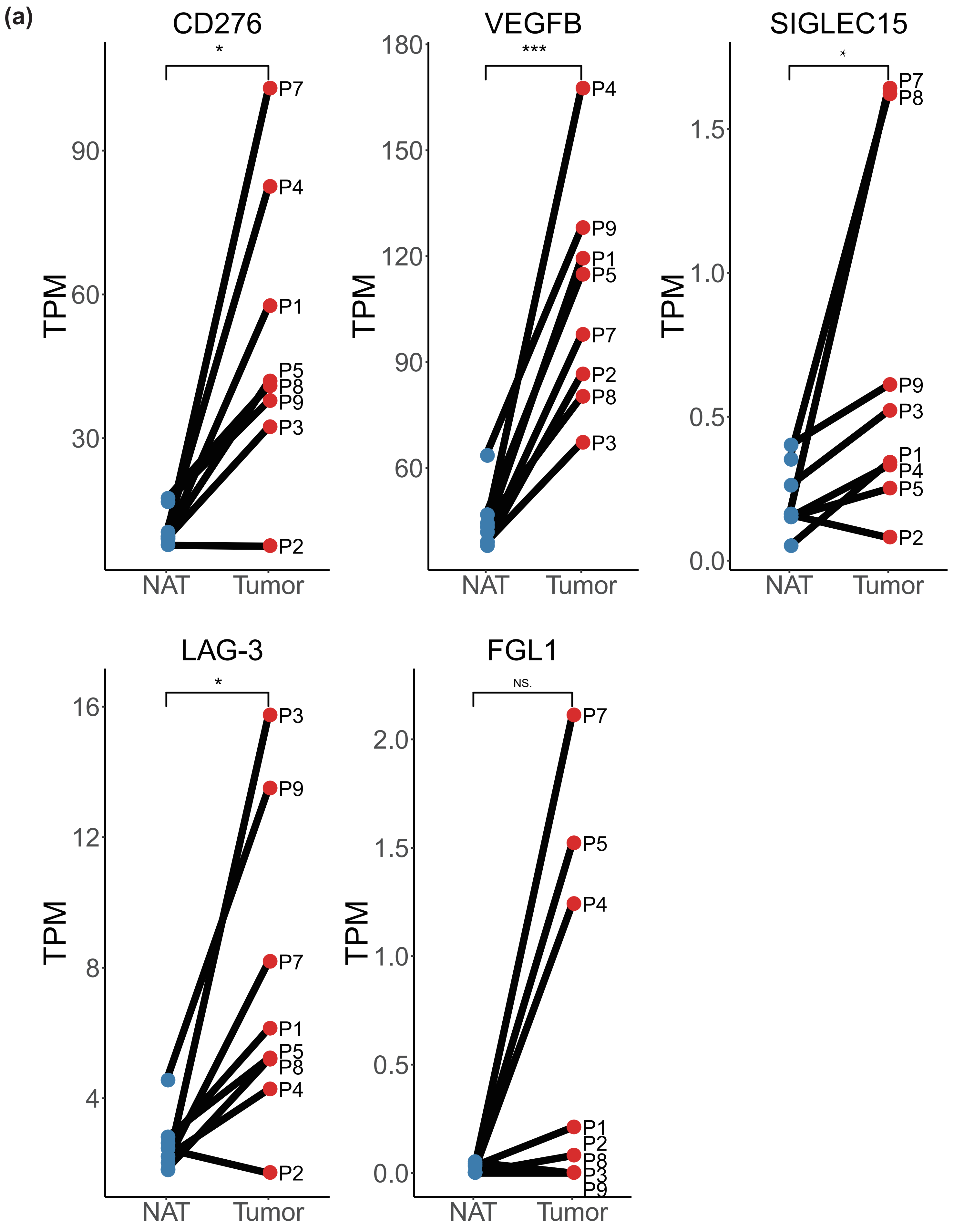

Supplementary figure 6

(a)

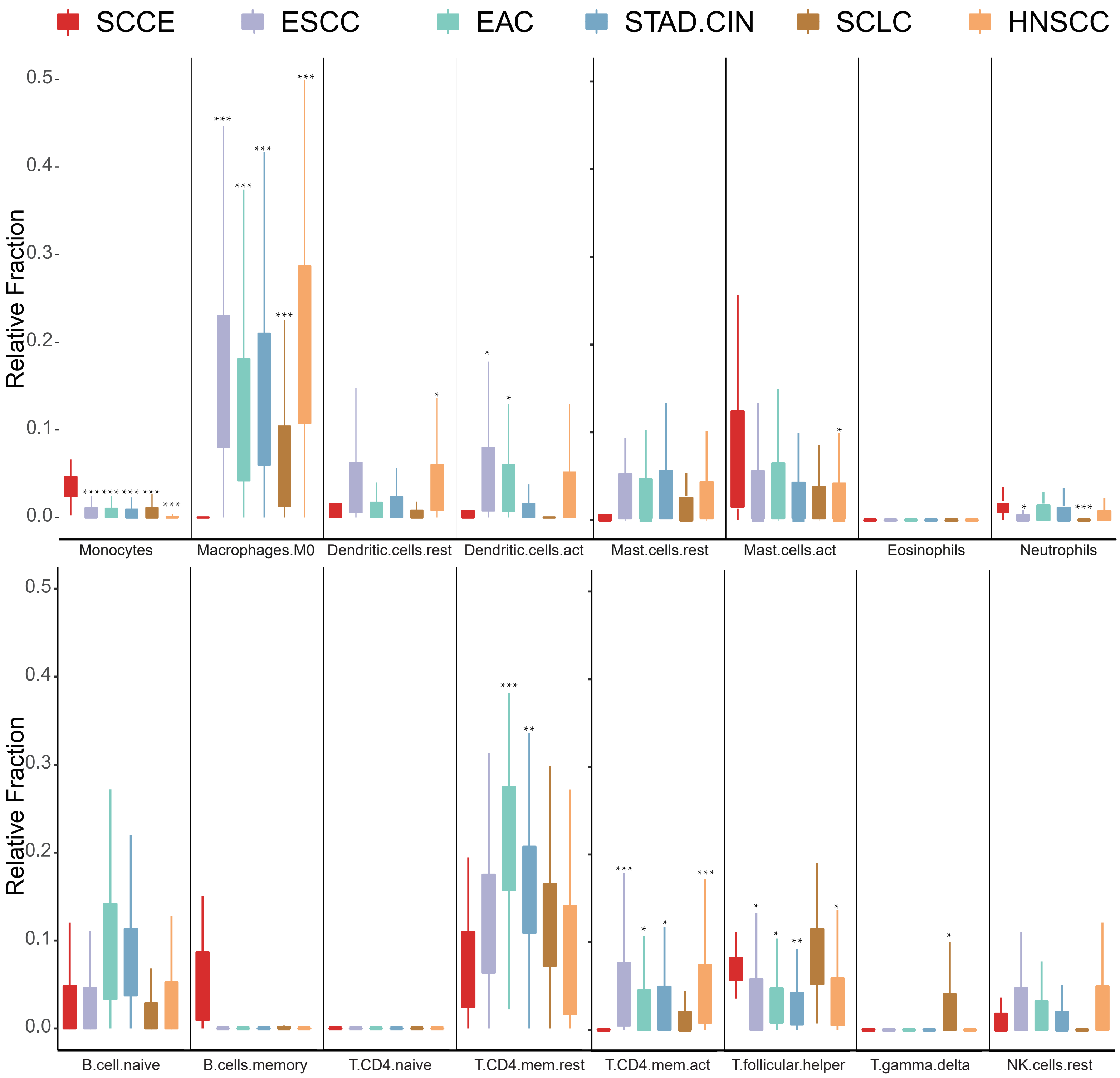

Supplementary figure 7

(a)

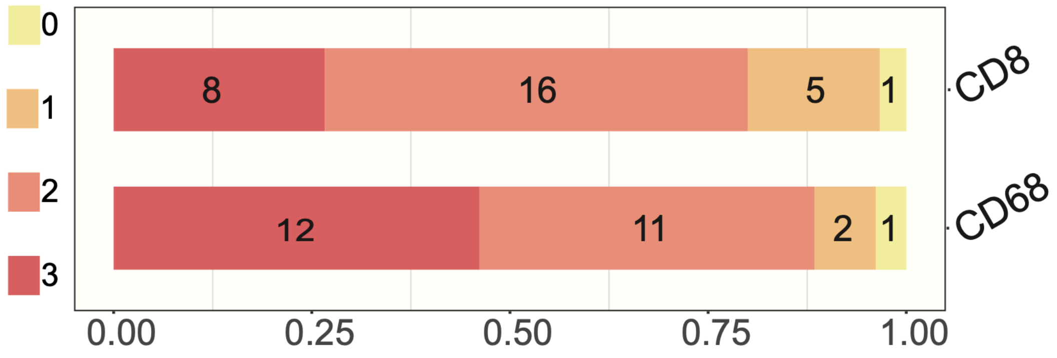

(b)

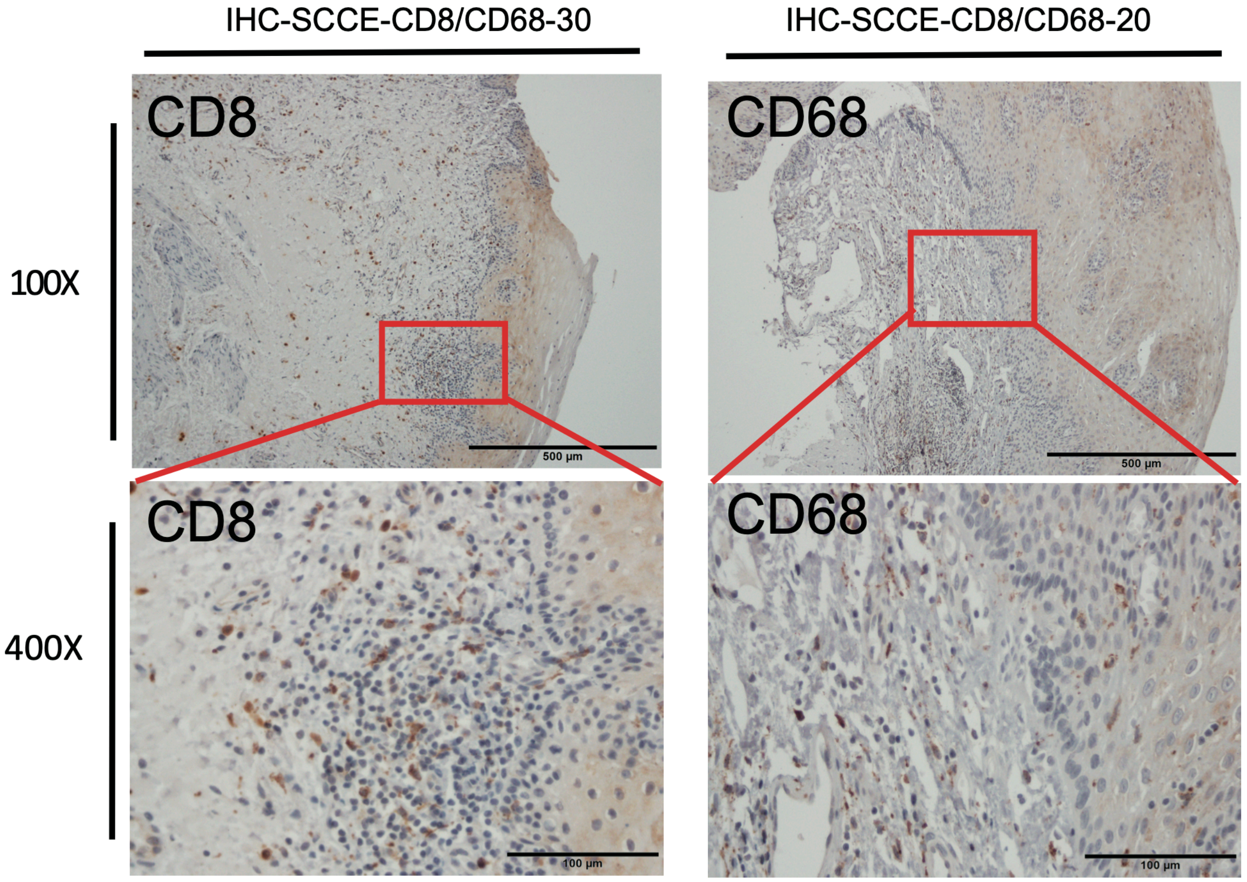

Supplementary figure 8

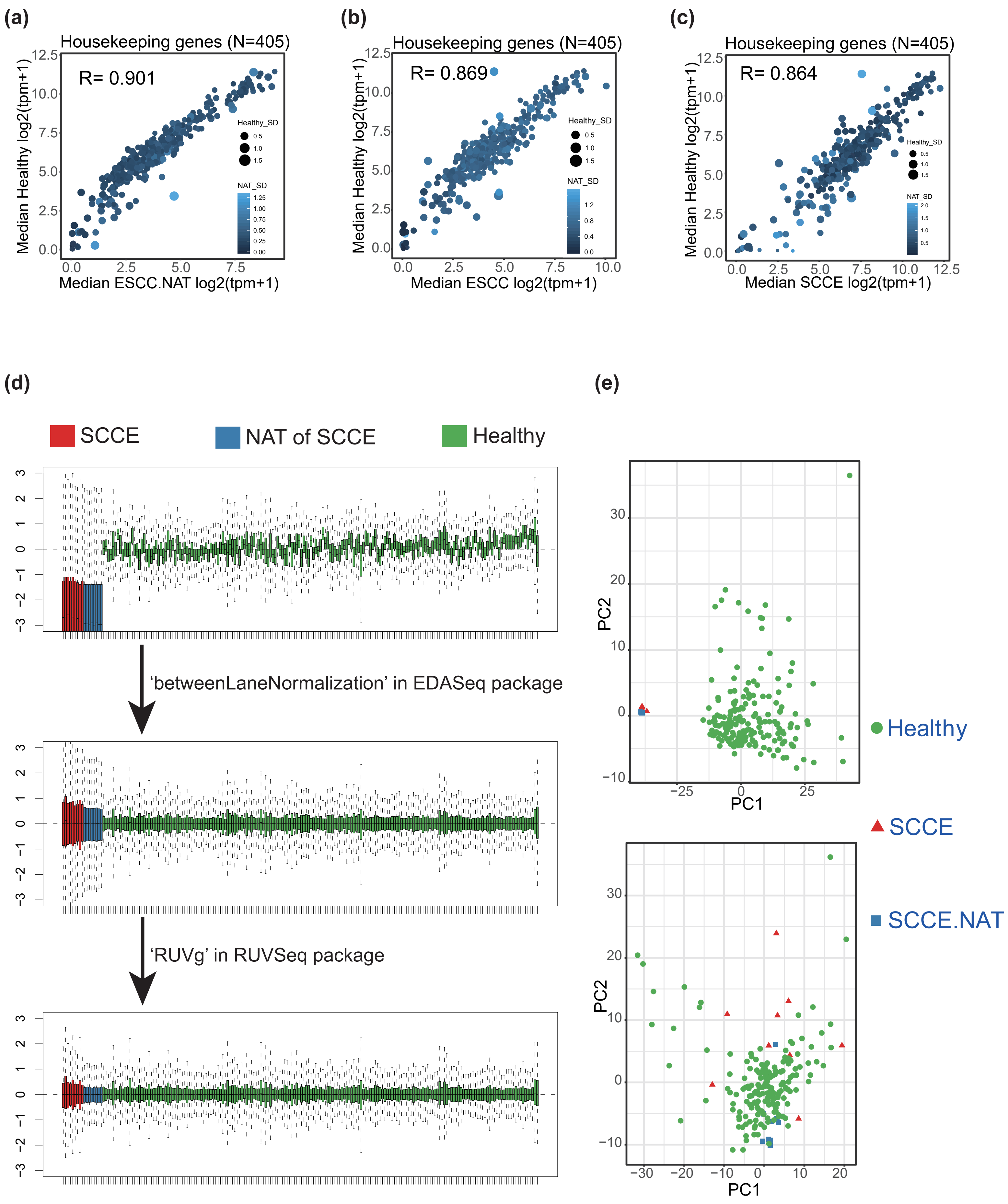

Supplementary figure 9

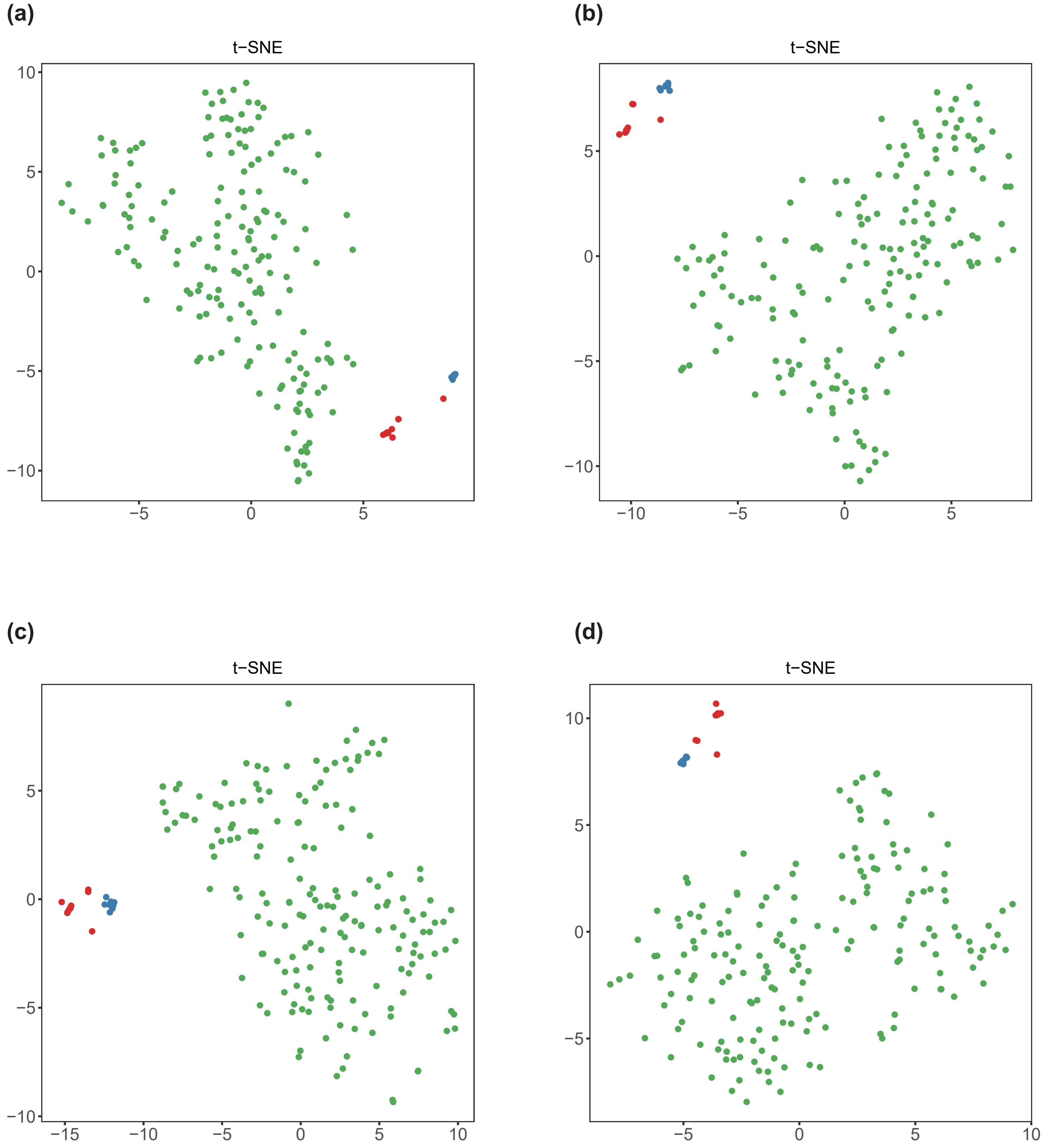

Supplementary figure 10

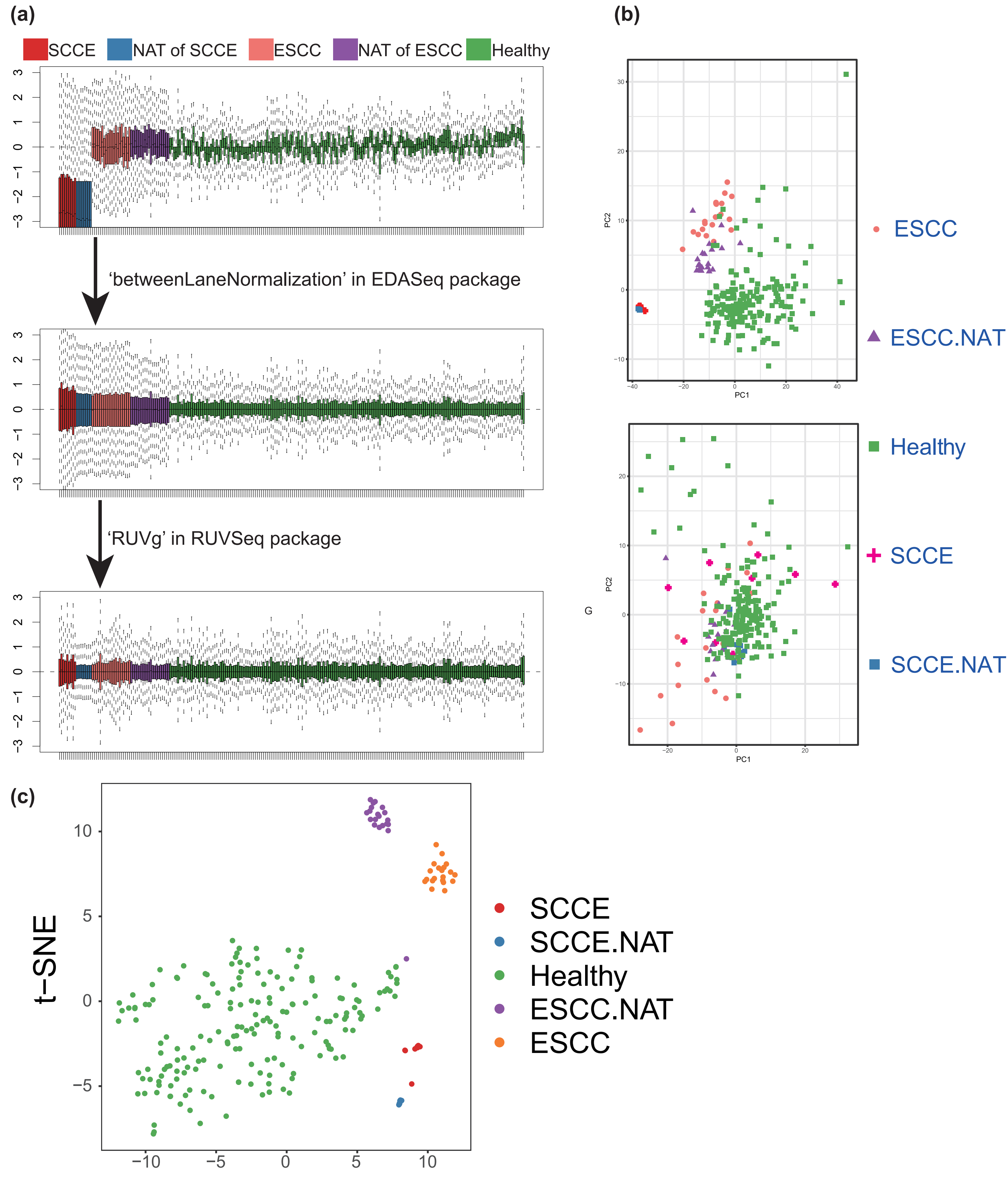

Supplementary figure 11

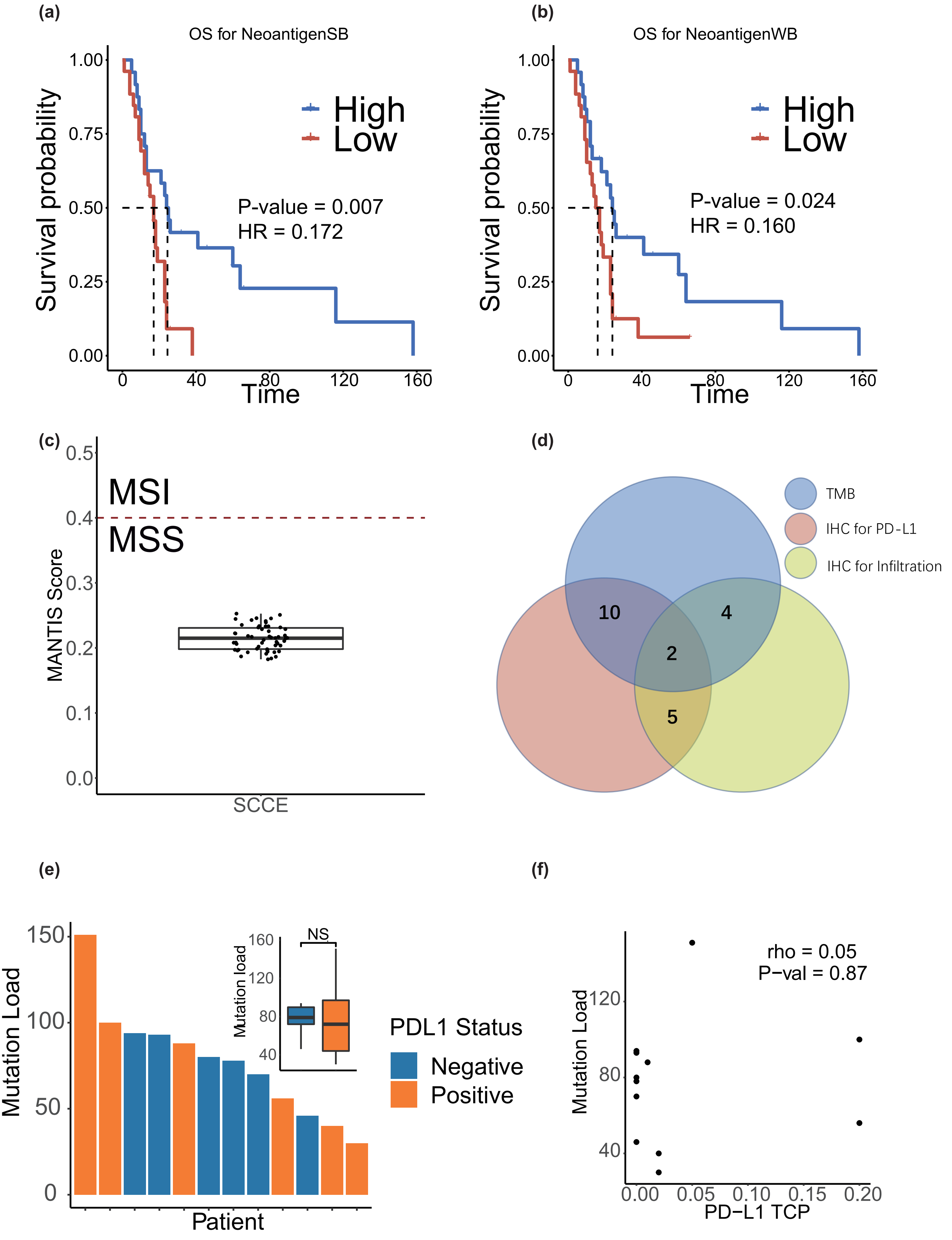

Supplementary Figure 12

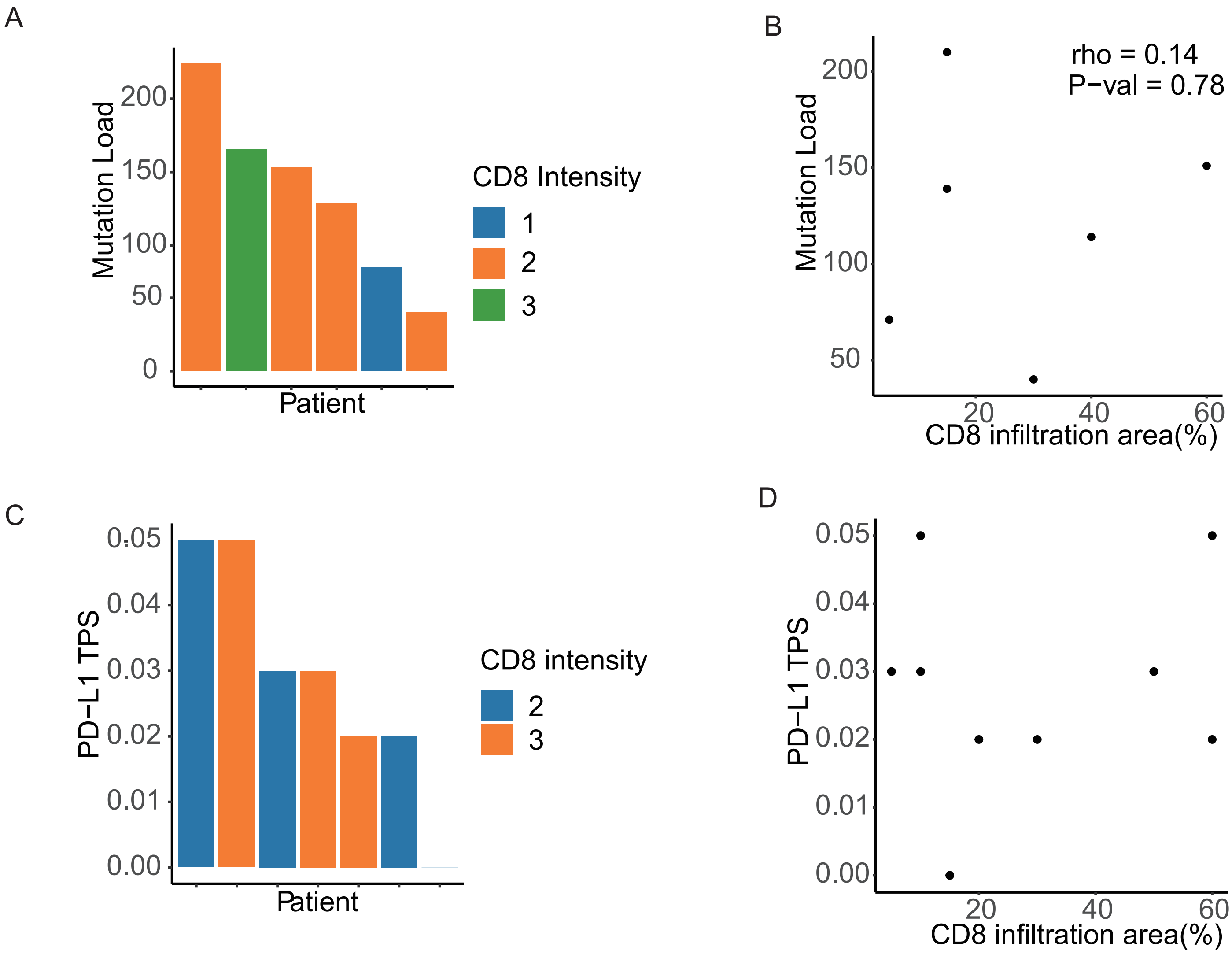

Supplement: Supplementary file 1 [file CTI2-9-e1173-s001.pdf]
